# Tuning of the RBR1-E2F/DP transcriptional module by the F-box protein FBL17

**DOI:** 10.1126/sciadv.adz2439

**Published:** 2026-02-18

**Authors:** Juliette Espanet, Xiaoning He, Ting Pan, Naomie Gentric, Thomas Potuschak, Bénédicte Desvoyes, Philippe Hammann, Johana Chicher, Rim Brik, Esther Lechner, David Latrasse, Aladár Pettkó-Szandtner, Crisanto Gutierrez, Cécile Raynaud, Zoltán Magyar, Moussa Benhamed, Shunping Yan, Sandra Noir, Pascal Genschik

**Affiliations:** ^1^ Institut de Biologie Moléculaire des Plantes, CNRS, Université de Strasbourg, 12, rue du Général Zimmer, 67084 Strasbourg, France.; ^2^ Université Paris-Saclay, CNRS, INRAE, Université Evry, Institute of Plant Sciences Paris-Saclay (IPS2), 91190 Gif-sur-Yvette, France.; ^3^ Hubei Hongshan Laboratory, Huazhong Agricultural University, 430070 Wuhan, China.; ^4^ Centro de Biologia Molecular Severo Ochoa, CSIC-UAM, 28049 Madrid, Spain.; ^5^ Plateforme Protéomique Strasbourg Esplanade du CNRS, Université de Strasbourg, 67084 Strasbourg, France.; ^6^ Institute of Biochemistry, HUN-REN Biological Research Centre, Szeged, Hungary.; ^7^ Institute of Plant Biology, HUN-REN Biological Research Centre, Szeged, Hungary.

## Abstract

F-box proteins of SCF E3 ligases have been documented to control the abundance of numerous critical regulatory proteins. In *Arabidopsis*, one of them, F-BOX-LIKE17 (FBL17), stands out for playing a key role in DNA replication, DNA damage, and, more recently, for the control of cell size. *FBL17* null mutants exhibit severe cellular defects leading to lethality. However, the molecular mechanisms by which FBL17 operate remain poorly understood. Here, we show that FBL17 interacts with different components of the RETINOBLASTOMA-RELATED1/E2F module and is involved in the protein turnover of E2Fa and E2Fb. However, mutations in *E2Fa* or *E2Fb* do not alleviate the severe *fbl17* phenotype but worsen it. By contrast, it is the accumulation of the transcriptional repressor E2Fc that causes *fbl17* mutant lethality. Our results highlight a key role for FBL17 in modulating the transcriptional control of E2F target genes ensuring precise control of cell cycle progression and avoiding uncontrolled DNA damage response.

## Teaser

FBL17 regulates the RBR1–E2F/DP transcriptional module to prevent aberrant activation of the DNA damage response in *Arabidopsis*.

## INTRODUCTION

In all eukaryotic organisms, the cell cycle is divided in four phases, with a first G_1_ gap phase, followed by S phase during which cells synthesize a complete copy of their DNA, a second gap G_2_ phase, and lastly mitosis (M) allowing sister chromatid separation and cytokinesis (*1*, *2*). Cell cycle progression is dependent on the activity of cyclin-dependent kinases (CDKs), which form complexes with cyclins, their regulatory subunits (*3*). In plants, this activity is conferred by A- and B-type CDKs, which are activated by different cyclins, expressed periodically during the cell cycle (*4*). It has been shown that CDKA;1, the only PSTAIRE CDK in flowering plants, is the main regulator of the G_1_/S transition, whereas a small family of CDKBs, specifically expressed during G_2_/M, is necessary for mitosis progression (*5*). The temporal activation and inactivation of CDKs during the cell cycle need to be regulated. While the T-loop phosphorylation of CDKA;1 is required for its activity, the plant cell cycle does not depend on an inhibitory phosphorylation during G_2_ (*4*). However, to restrain CDK activity, especially at the exit of mitosis and in early G_1_, all eukaryotic cells take advantage of CDK inhibitor (CKI) proteins that either interact with the CDK to prevent cyclin binding or associate with the whole cyclin-CDK complex to inactivate it (*6*). In plants, two classes of CKIs, called KIP-RELATED PROTEINS (KRPs; KRP1-7 in *Arabidopsis*) and SIAMESE-RELATED proteins (SMRs) have been characterized (*7*, *8*). It is believed that KRPs mainly inhibit CDKA;1-CYCD complexes during G_1_, whereas members of the SMR family bind to both CDKA;1-CYCD and M-phase CDK complexes and are important for the regulation of endoreplication (*9*, *10*). To enter S phase, cells need to decrease the level of CKI proteins to release CDK activity. In both yeast and metazoans, this is at least in part achieved by the degradation of CKIs through the ubiquitin proteasome system. This pathway depends on ubiquitin ligases (E3) that together with ubiquitin-conjugating enzyme (E2) transfer ubiquitin (Ub) to a substrate protein and, depending on the topology of the Ub chain, can lead to its degradation via the 26*S* proteasome (*11*). In budding yeast, to enter S phase, the CKI called Sic1 is first phosphorylated by G_1_ cyclin-CDK activity to be ubiquitylated by an SCF (Skp1–Cullin–F-box-protein) type of E3 Ub ligase (*12*, *13*). Note that SCF complexes are multisubunit Ub ligases with F-box proteins acting as specificity determinants by binding to their substrates (*14*). This mechanism is conserved, and in mammals, the leucine-rich repeat–type F-box protein Skp2, as part of the SCF^Skp2^ complex, also ubiquitylates the phosphorylated forms of the CKI p27/Kip1 to direct its proteasomal degradation and to promote S phase entry (*15*–*17*).

Among the large number of *Arabidopsis* F-box proteins (*18*), FBL17 appears as the closest functional homolog of the mammalian Skp2 (*19*). *FBL17* was identified as an essential gene needed for male germ cell division (*20*, *21*), but viable *Arabidopsis fbl17* null mutant plants can be recovered at very low frequency and they revealed major cell cycle defects such as impaired entry and/or progression in S phase and the suppression of endoreplication (*22*). This phenotype can potentially be explained by a high accumulation of CKIs, as overexpression of KRPs in plants can block S-phase CDK activity (*23*) and is also consistent with the phenotypic resemblance of *fbl17* with the *cdka;1* null mutant (*22*, *24*). Moreover, the steady-state protein level of KRP2 was found increased in *fbl17* null mutants (*22*), and it was recently found that *FBL17* is required to destroy KRP4 to maintain cell size homeostasis (*25*). However, the *FBL17* loss-of-function phenotype cannot be explained solely by the failure to degrade KRP proteins. For instance, the transcriptome of *fbl17* mutant plants showed a strong up-regulation of numerous cell cycle and DNA damage genes (*26*), which has not been observed for strong KRP2-overexpressing lines. The mutation of *SOG1*, encoding a master transcriptional regulator of the DNA damage response (DDR) (*27*), did not suppress the constitutive activation of the genes linked to DNA damage in *fbl17* (*26*). Nonetheless, many of these genes were predicted to be targets of the RETINOBLASTOMA-RELATED 1 (RBR1)–E2Fa transcriptional regulatory pathway. Hence, RBR1 directly represses a number of genes involved in DNA damage, notably through the E2Fa transcription factor (*28*–*30*).

In *Arabidopsis*, there are three canonical E2F transcription factors, E2Fa, E2Fb, and E2Fc, that form dimers with one of the two dimerization partner proteins (DPa and DPb). It was proposed that E2Fa and E2Fb are transcriptional activators, whereas E2Fc would act as a transcriptional repressor of cell proliferation (*31*). However, biochemical and genetic studies revealed more complex regulatory roles of these factors (*32*–*35*). In proliferating cells, E2Fa can stimulate the G_1_/S transition, but in complex with RBR1, it functions as a repressor of endocycle and differentiation (*36*). E2Fb, which is expressed constitutively throughout the cell cycle, appears to stimulate both G_1_/S and G_2_/M-phase transitions (*37*). Although E2Fb can also form a repressive complex with RBR1 that inhibits cell proliferation and induces quiescence, leading to tissue differentiation during leaf development (*38*). The role of E2Fc is less understood, but it was reported to act mainly as a transcriptional repressor of cell proliferation and an activator of endoreplication in differentiated cells (*39*, *40*). Moreover, E2Fc, as well as E2Fb, RBR1, and MYB3Rs, but not E2Fa, is part of multiprotein complexes, so called DREAM-like (DP, RBR1, E2F, and MYB) complexes, implicated in the repression of cell cycle genes (*41*, *42*).

Previous works highlighted an intricate relation between FBL17 and the RBR1-E2F pathway. On one hand, *FBL17* expression is transcriptionally regulated by E2Fa and DPa and repressed by RBR1 (*20*, *43*). On the other hand, as indicated above, many genes involved in cell cycle and DNA damage known to be transcriptional targets of E2Fs were found up-regulated in *fbl17* knockout mutants (*22*, *26*). Here, we investigated the genetic and molecular interactions between FBL17 and the canonical E2F transcription factors. Our work sheds light on the function of FBL17 and identified E2Fc as a causal factor of the strong cellular and molecular phenotype triggered by the *fbl17* mutation.

## RESULTS

### The severe *FBL17* loss-of-function phenotype is suppressed by the *e2fc* mutation

**Fig. 1.**
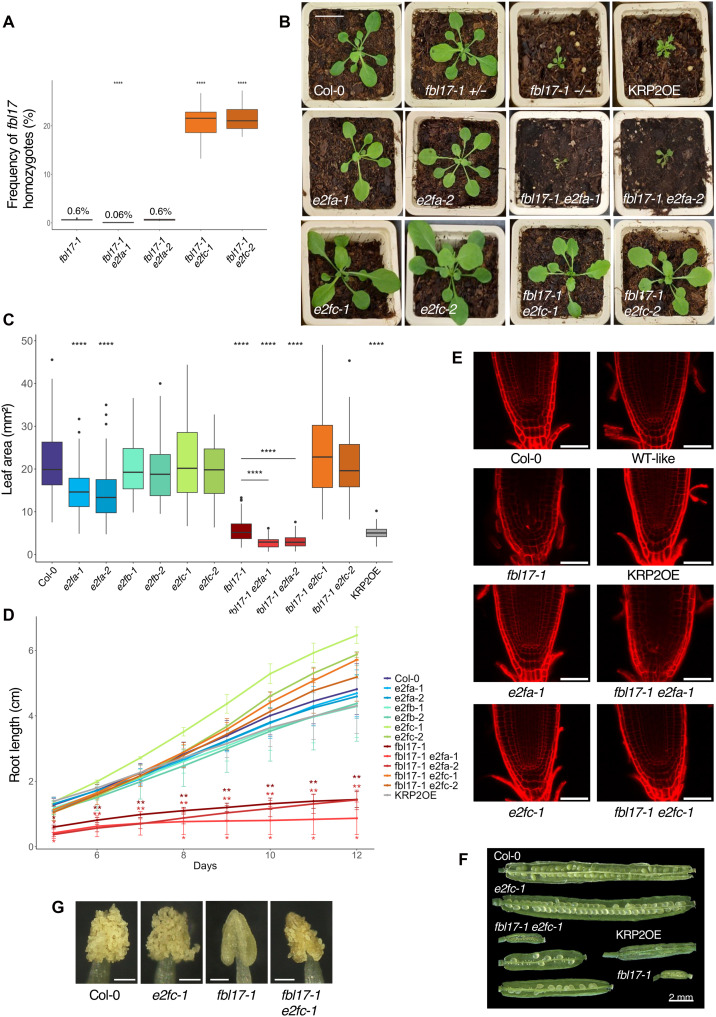
The *e2fc*, but not the *e2fa* mutation, suppresses *fbl17* developmental defects. (**A**) Frequency of *fbl17* homozygotes plants (in percentage) in the progeny of the indicated genotypes. Seedlings were grown in vitro. Asterisks indicate significant difference between genotypes and *fbl17-1* for three biological replicates: ****P < 0,0001 by Wilcoxon-Mann-Whitney test [4 < *N* (per genotype) < 25]. Complete statistical analyses are given in table S1A. (**B**) Representative pictures of 4-week-old *Arabidopsis* rosettes for the different genotypes. Scale bar, 2 cm. (**C**) Quantification of leaf area (in square millimeters) of the first pair of leaves from 20-day-old plants of the indicated genotypes grown under in vitro conditions. Asterisks indicate significant difference between the different genotypes and Col-0 plants from three biological replicates: ****P < 0.0001 by Wilcoxon-Mann-Whitney test [35 < *N* (per genotype) < 169]. Complete statistical analyses are given in table S1B. (**D**) Root length (in centimeters) measured from 5-day-old in vitro grown plants for 7 days. Error bars indicate SE variation from at least three biological replicates. Asterisks indicate significant difference between the different genotypes and Col-0 plants from at least three biological replicates: *P < 0.05 and **P < 0.01 by Wilcoxon-Mann-Whitney test [25 < *N* (per genotype) < 106]. Complete statistical analyses are given in table S1C. (**E**) RAM organization of the indicated genotypes. Representative images of root tips of 7-day-old seedlings grown in vitro before propidium iodide staining. Scale bars, 50 μm. (**F**) Open siliques of the indicated genotypes, including three different *fbl17-1 e2fc-1* siliques illustrating phenotypic variations. (**G**) Representative pictures of anthers from unopened flowers for the indicated genotypes. Scale bars, 100 μm.

*Arabidopsis fbl17* null mutant plants exhibit severe developmental and cellular defects associated with the misexpression of numerous cell cycle and DDR genes (*26*). To investigate the possible contribution of the RBR1-E2F pathway in the misexpression of these genes, we have examined the genetic interactions between *FBL17* and the three canonical *E2F* genes. For this, two different alleles of each *e2fa*, *e2fb*, and *e2fc* mutant (fig. S1) were introgressed into the *fbl17*-*1* mutant background (*22*). Note that given the sterility of *fbl17* homozygous mutants, the seed populations were maintained in the *fbl17* heterozygous background (*fbl17+/−*), and the segregating wild-type (WT)–like plants (i.e., *fbl17+/−* or *fbl17+/+*) were used as control for some experiments. In addition, the KRP2OE line (*22*) was used to monitor effects solely due to KRP overaccumulation. A first observation was that double homozygous mutant *fbl17 e2fb* seedlings were never identified in the progeny populations of both *fbl17-1 e2fb-1* and *fbl17-1 e2fb-2* crosses [with more than 160 and 180 seedlings polymerase chain reaction (PCR)–screened for each genotype], suggesting a possible synthetic lethality as a result of the deletion of both genes. Conversely, we were able to identify *fbl17 e2fa* double mutant plants. The frequency of *fbl17* homozygous escapers was below 1% as previously reported (*22*), and this is also what we observed for *fbl17-1 e2fa-2* mutants, while the frequency of the *fbl17-1 e2fa-1* double mutants was even 10 times less (Fig. 1A). In addition, the *fbl17-1 e2fa-1* mutant presents an even stronger macroscopic phenotype than the single *fbl17* mutant, both on soil and upon in vitro culture (Fig. 1B and fig. S2A), and most of these plants did not survive. On the contrary, the macroscopic phenotype of *fbl17-1 e2fa-2* double mutant at young developmental stages resembles *fbl17* (Fig. 1B), although a few weeks later, those mutant plants are bolting earlier than *fbl17-1* single mutants (fig. S2B). Measurements of the leaf area (first pair) and root length of in vitro grown plants showed that for both *fbl17 e2fa* double mutant combinations, organ size remained strongly affected (Fig. 1, C and D) and that the root apical meristem (RAM) disorganization previously reported for *fbl17* (*22*) is also maintained in *fbl17-1 e2fa-1* root tips (Fig. 1E). Notably, *fbl17 e2fc* double mutant plants could be recovered, and they present an obvious suppression of the *fbl17* macroscopic phenotype being more similar to WT Col-0 or *e2fc* single mutant plants (Fig. 1B). Moreover, up to 20% of double homozygous *fbl17 e2fc* seedlings could be identified in the progenies of both *fbl17-1+/− e2fc-1−/−* and *fbl17-1+/− e2fc-2−/−*, a ratio close to an expected Mendelian segregation (Fig. 1A). In addition, *fbl17 e2fc* plants are fertile and develop siliques containing few but viable seeds (Fig. 1F and fig. S3, A and B). Note that siliques of the double mutant present a variation in size with some very small seedless siliques, resembling homozygous *fbl17*, but also longer siliques with a variable number of viable seeds (fig. S3, A and B). This effect can be explained, at least in part, by the restored pollen production in the *fbl17 e2fc* anther (Fig. 1G). Despite a lower amount of observed *fbl17 e2fc* mutant grains, these appear well formed and fully viable (fig. S3C). In line with the macroscopic phenotype, quantifications of the leaf area (first pair) and the root length also showed the reestablishment of organ size in the double mutants to levels comparable to Col-0 and *e2fc* (Fig. 1, C and D). Moreover, the disorganized RAM phenotype of *fbl17-1* was suppressed by the *e2fc-1* mutation as observed by confocal microscopy (Fig. 1E).

**Fig. 2.**
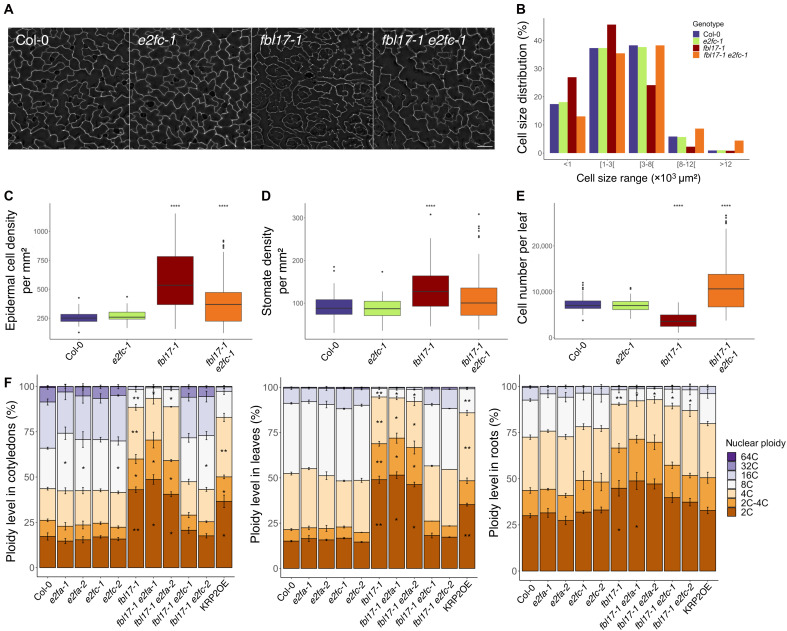
The *e2fc* mutation mostly suppresses the *fbl17* cellular phenotypes. (**A** to **E**) The data were obtained from cellular leaf imprints made on the adaxial epidermal cells of the first pair of leaves of 20-day-old plants of the indicated genotypes and further analyzed using the Fiji software (ImageJ 1.52p; http://imageJ.nih.gov/ij). (A) Representative pictures of nail polish cellular imprints observed using an optical microscope upon phase contrast conditions. Scale bar, 100 μm. (B) Cell size distribution (in square micrometers), where five size ranges are distinguished. (C) Epidermal cell density per square millimeters. (D) Stomatal cell density per square millimeters. (E) Cell number per leaf calculated using leaf size data and cell density (considering both pavement cells and stomata). Asterisks indicate significant difference between the different genotypes and Col-0 plants from three biological replicates: ****P < 0.0001 by Wilcoxon-Mann-Whitney test [71 < *N* (per genotype) < 155]. Complete statistical analyses are given in table S1 [(D) to (F)]. (**F**) Flow cytometry analyses of the nuclear DNA content frequency in cotyledons, leaves, and roots of 20-day-old plants grown in vitro. Nuclear ploidy levels between 2C and 4C (orange-brown) correspond to proliferating cells, and nuclear ploidy levels between 8C and 64C (purple) correspond to endocycling cells. At least 10,000 nuclei were analyzed for each sample, and error bars represent SE variation between the sample of the same genotype from at least three biological replicates [10 < *N* (per genotype) < 42 for leaves, 13 < *N* (per genotype) < 35 for cotyledons, and 12 < *N* (pergenotype) < 38 for roots]. Complete statistical analyses are given in tables S1 [(G) to (I)].

Next, we examined whether the rescue of the *fbl17* phenotype by the *e2fc* mutation could be explained by the restoration of either cell proliferation, cell growth, or both. Cell imprints were made from adaxial leaf epidermis to quantify pavement cell size and number (Fig. 2, A to E). While Col-0 and *e2fc-1* cells appear similar and present the classic *Arabidopsis* jigsaw shape of epidermal cells, *fbl17-1* cells are smaller and their shape more roundish (Fig. 2, A and B). The *fbl17-1 e2fc-1* cells exhibit an intermediary phenotype with an apparent rescue of cell size associated with an attenuated jigsaw shape. More precisely, by establishing the distribution of cell sizes (Fig. 2B), it appears that the *fbl17-1 e2fc-1* mutant exhibits less cells of small size (i.e., <1 × 10^3^ μm^2^) but more cells of bigger size (i.e., above 8 × 10^3^ μm^2^) compared with *fbl17-1*. Then, by combining epidermal cell density and leaf area, the cell number per leaf could be estimated in the different genotypes (Fig. 2, C to E). This revealed that *fbl17-1 e2fc-1* mutant plants did not only rescue the cell number compared to *fbl17-1* but also have more cells in average than Col-0 and *e2fc-1*. Together, we conclude that the *fbl17 e2fc* mutant line presents larger cells and a higher number of cells compared to *fbl17*, broadly suppressing cell elongation and cell proliferation defects of this mutant.

Endoreplication in plants is generally associated to cell differentiation, and in some tissues, like epidermal pavement cells, an increase of the ploidy level can be correlated with increased cell size (*44*). Thus, we measured nuclear DNA contents from mature cotyledons, leaves, and roots of the different genotypes and calculated the endoreplication index (Fig. 2F and fig. S4).Compared to *fbl17-1* nuclei that present a very strong reduction in ploidy levels as previously reported (*22*), flow cytometry data show that ploidy levels in *fbl17-1 e2fc-1* cotyledons and leaves and, to a lesser extent, in roots were restored to a level comparable to Col-0 (Fig. 2F and fig. S4). We also verified trichome branching, as the branch number of these cells is typically correlated with their ploidy level (*44*). In accordance with a defect in endoreplication, *fbl17* trichomes are mono-branched instead of being three-branched as in the WT (*22*). The *fbl17-1 e2fc-1* mutants present numerous trichomes with two branches (fig. S2C), suggesting a partial reestablishment of endoreplication in those cells. Regarding the *fbl17 e2fa* plants, mutation of *e2fa* was unable to suppress the endoreplication defect in *fbl17* cotyledons, leaves, and roots (Fig. 2F and fig. S4), and *fbl17-1 e2fa-1* trichomes are mono-branched, like in *fbl17* (fig. S2C). Overall, despite some cellular differences exhibited by both *fbl17-1 e2fc-1* and *fbl17-1 e2fc-2* allele combinations as indicated above, our results identified *e2fc* as the first mutation being able to revert the severe phenotype of an *FBL17* loss-of-function mutant.

### The misexpression of cell cycle and DDR genes in *fbl17* is markedly attenuated by the *e2fc* mutation

**Fig. 3.**
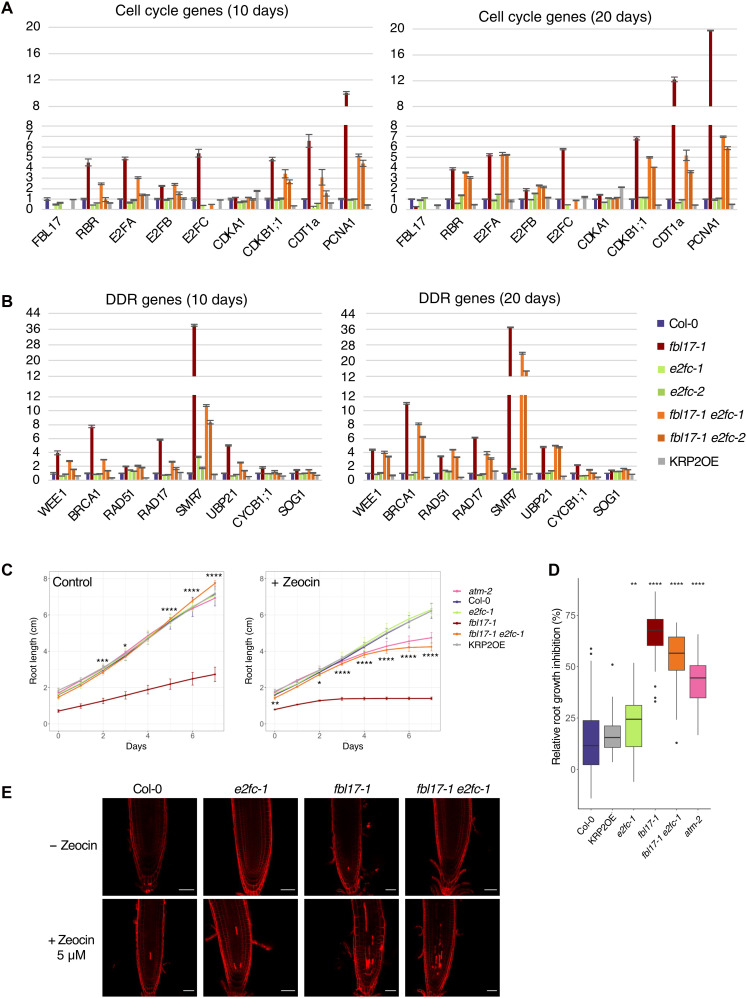
The misexpression of numerous cell cycle and DDR genes is attenuated in the *fbl17 e2fc* double mutant. (**A** and **B**) Relative expression levels of (A) cell cycle and (B) DDR genes analyzed by RT-qPCR. Transcripts were extracted from 10-day-old (left) and 20-day-old (right) whole seedlings grown in vitro, and error bars represent variation within three technical replicates. The experiment was repeated in three biological replicates and gave the same tendency. (**C**) Root growth elongation of the indicated genotypes in 5-day-old seedlings grown under standard conditions and then transferred onto control medium (left) or medium containing 5 μM zeocin (right) for further 7 days of culture. Asterisks indicate significant difference between the different genotypes and Col-0 plants from three biological replicates: *P < 0.05, **P < 0.01, ***P < 0.001, and ****P < 0.0001 by Wilcoxon-Mann-Whitney test [32 < *N* (per genotype) < 90 under standard conditions, 35 < *N* (per genotype) < 84 under zeocin]. Complete statistical analyses are given in table S1J. (**D**) Percentage of root length inhibition for the experiment described in (C). Root length inhibition percentage was calculated for each root using the formula: 100 − [100 × (d12 length − d5 length)]/d12 final length mean. The mean percentage was then calculated for each genotype. Asterisks indicate significant difference between the different genotypes and Col-0 plants from three biological replicates: **P < 0.01 and ****P < 0.0001 by Wilcoxon-Mann-Whitney test [35 < *N* (per genotype) < 84]. Complete statistical analyses are given in table S1K. (**E**) Representative images of root tips of 5-day-old seedlings transferred onto control medium or medium containing 5 μM zeocin for further 3 days of growth before propidium iodide staining. Scale bars, 50 μm.

By performing an RNA sequencing (RNA-seq) analysis, we previously reported that about 25% of the *Arabidopsis* genome is differentially expressed (DE) in *fbl17* and that a large number of cell cycle and DDR genes were up-regulated (*26*). We next performed quantitative reverse transcription PCR (RT-qPCR) for a set of these genes in the different genotypes of 10-day-old and 20-day-old seedlings (Fig. 3, A and B). While 10-day-old seedlings contain still many proliferating cells, in 20-day-old plants, cells are differentiated and mostly expanding, partially through the endocycle (*45*). In accordance with our previous data (*22*), cell cycle or DDR gene expression was not substantially altered in the KRP2OE line despite its strong phenotype on plant growth (Fig. 1), but these genes were greatly up-regulated in *fbl17* mutant plants (Fig. 3, A and B). The overexpression of cell cycle genes observed in *fbl17* was found significantly attenuated in the *fbl17 e2fc* double mutant. However, the decrease in the expression level was variable depending on the gene investigated and the plant developmental stage (Fig. 3A). Thus, the expression level of genes such as *CDT1a* and *PCNA1* exhibited a strong down-regulation, whereas *RBR1*, *E2Fa*, and *CDKB1;1* remained still overexpressed especially in 20-day-old plants. A similar trend was observed with genes involved in DDR (Fig. 3B). Hence, the expression of genes involved in DNA repair [*RAD17* and *BRCA1*; (*46*)] or DDR signaling [*CYCB1;1*, *WEE1*, *SMR7*, and *UBP21*; (*28*, *47*)] that is up-regulated in *fbl17* was greatly attenuated by the *e2fc* mutation in 10-day-old and, to a lesser extent, in 20-day-old plants.

As the *fbl17* mutant is hypersensitive to double-strand break (DSB) DNA damages (*26*), we measured the response of the *fbl17 e2fc* double mutant to zeocin (Fig. 3, C and D). As ataxia-telangiectasia mutated is a key kinase in the DSB repair pathway (*48*), we used the *atm-2* mutant that serves as a hypersensitivity benchmark. Despite a notable reduction in the expression of DDR genes (Fig. 3B), the *fbl17 e2fc* double mutant still remained hypersensitive to the treatment at a level similar to the *atm-2* hypersensitive control. The *fbl17 e2fc* mutant also exhibits increased DSB-induced cell death, but contrary to *fbl17*, it did not show constitutive cell death in the absence of zeocin (Fig. 3E). These results suggest that the global suppression of *fbl17* developmental and cellular defects, including constitutive cell death, by the *e2fc* mutation could result from an overall attenuation of both cell cycle and DDR gene misexpression, although other scenarios cannot be excluded.

### Components of the RBR1/E2F module are part of the FBL17 interactome

**Fig. 4.**
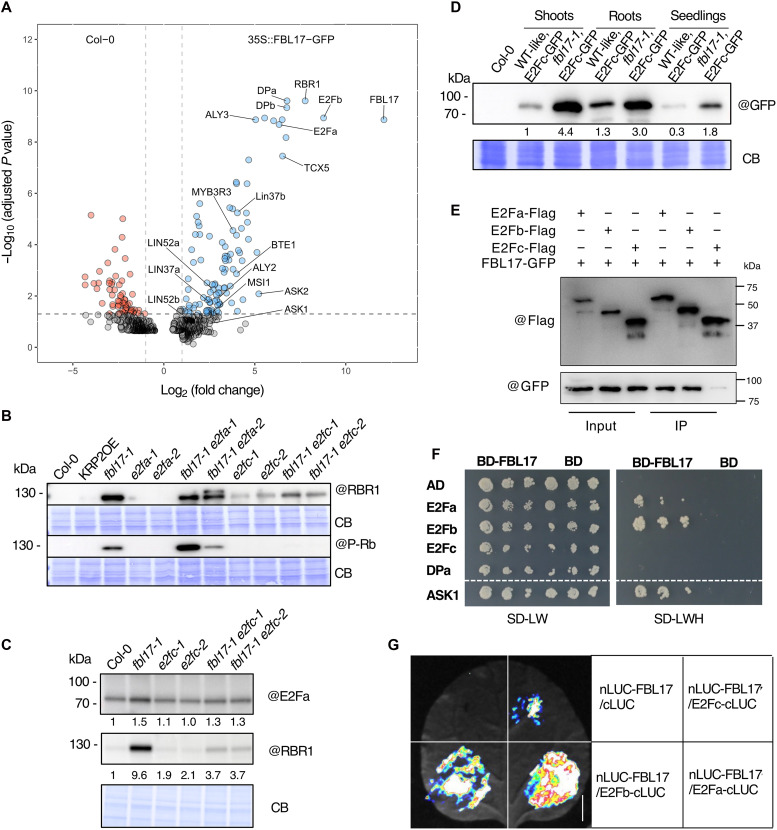
FBL17 interacts with components of the RBR1/E2F module. (**A**) Proteins statistically enriched after IP with FBL17-GFP and identified by mass spectrometry. Proteins from the four FBL17-GFP biological replicates are represented in a volcano plot (Col-0/35S:FBL17-GFP). Statistical analyses were performed on Prostar with a LIMMA test and a Benjamini-Hochberg validation (adjusted *P* value). The vertical dotted lines show large-scale changes in FC (*x* axis, FC < 0.5 left, FC > 2 right), and the horizontal dotted line shows high statistical significance (*y* axis, adjusted *P* < 0.05). (**B**) Immunoblot analysis of protein extracts from 10-day-old seedlings of the indicated genotypes to reveal total and phosphorylated RBR1 protein levels. CB, Coomassie blue staining. (**C**) Immunoblot analysis of protein extracts from 20-day-old seedlings of the indicated genotypes to monitor E2Fa and RBR1 protein levels. (**D**) Immunoblot analysis of protein extracts from 10-day-old seedlings of pE2Fc:E2Fc-GFP in WT-like and the *fbl17* background to reveal E2Fc-GFP protein level. Shoots and roots have also been analyzed separately. (**E**) In Co-IP assays, E2Fa-Flag, E2Fb-Flag, or E2Fc-Flag was coexpressed with FBL17-GFP in *Arabidopsis* protoplasts. The protein samples were immunoprecipitated by GFP-Trap. Both the input and immunoprecipitated (IP) samples were subjected to Western blotting using anti-GFP or anti-FLAG antibodies. (**F**) Yeast two-hybrid interactions. FBL17 was tested pairwise with the three *Arabidopsis* E2Fs and DPa. ASK1 was used as a positive control. Growth on selective plates without leucine, tryptophan, and histidine (SD-LWH) and on control plates without leucine and tryptophan (SD-LW) is shown. BD and AD stand for DNA-binding domain and activating domain, respectively. Pictures of the plates were taken after 4 days at 30°C. (**G**) In split luciferase assays, the E2Fa, E2Fb, or E2Ffc was fused to cLUC, and FBL17 was fused to nLUC. The proteins were transiently expressed in *N. benthamiana*. The luminesce detected by a CCD camera indicates interaction. Scale bar, 3 cm.

To further explore the molecular function of FBL17, we undertook a nonbiased immunoprecipitation (IP) coupled with mass spectrometry approach, to unravel the interactome of the F-box protein using the previously reported 35S:FBL17-GFP line (*22*). This analysis was performed with 10-day-old seedlings grown in vitro and containing actively dividing cells. Proteins significantly enriched among the FBL17 immunoprecipitated proteins were highlighted by a statistical analysis, calculating normalized fold changes (FCs) and adjusted *P* values (Fig. 4A and table S2). As expected, we identified ASK1 and ASK2 proteins, two orthologs of the SCF core subunit Skp1. Unexpectedly, no KRP protein was found statistically enriched among FBL17 immunoprecipitated proteins. Instead, we identified the cell cycle regulators RBR1, E2Fa, and E2Fb and both DPa and DPb proteins as predominantly enriched in the FBL17 protein interactome. The interaction between FBL17 and RBR1 was already reported in cisplatin-treated cell culture (*41*). Moreover, we also found several components of the DREAM-like complex (*41*, *42*), including LIN37a/b, LIN52a/b, ALY2/3, TCX5/LIN54a, and MYB3R3 among others (Fig. 4A and table S2). This result suggests that FBL17 may directly regulate the RBR1-E2F/DP module and potentially the DREAM complex at the posttranslational level, not only upon genotoxic stress condition.

Next, we investigated the protein steady-state levels of RBR1 and E2Fa, as specific antibodies were available. For RBR1, we observed a strong accumulation of the protein in the *fbl17* single mutant compared to Col-0 plants (Fig. 4, B and C). Notably, the use of an antibody that specifically recognizes an RBR1 phosphorylated form also showed a higher accumulation of the modified form of the protein in this mutant. In *fbl17 e2fa* double mutants, the higher steady-state level of RBR1 was maintained to a similar extent as in *fbl17*. Conversely, in *fbl17 e2fc* double mutants, RBR1 protein accumulation was significantly lower than in *fbl17*, and the phosphorylated form of RBR1 was not detected anymore (Fig. 4B). To a lesser extent, we also observed a higher accumulation level of the E2Fa protein level in the *fbl17* single mutant compared to Col-0 plants (Fig. 4C), although this effect was more pronounced in younger (10-day-old) seedlings (fig. S5). Last, we wondered about the accumulation level of the E2Fc protein in the *fbl17* mutant. Since no E2Fc antibody is available, we used an E2Fc reporter line expressing an E2Fc–green fluorescent protein (GFP) fusion protein under the control of its endogenous promoter (*49*). We crossed the pE2Fc:E2Fc-GFP reporter line with the *fbl17-1* heterozygote mutant and selected double homozygotes for both the mutation and the transgene. Similarly to RBR1, the level of the E2Fc-GFP fusion protein was found greatly increased in *fbl17* null mutant (Fig. 4D).

Notably, some differences in E2Fc-GFP accumulation could be observed between shoots and roots of *fbl17-1*. Whether FBL17 may act in a tissue-specific or context-dependent manner would require further investigations.

### FBL17 directly interacts with E2Fa and E2Fb and is involved in their protein turnover

**Fig. 5.**
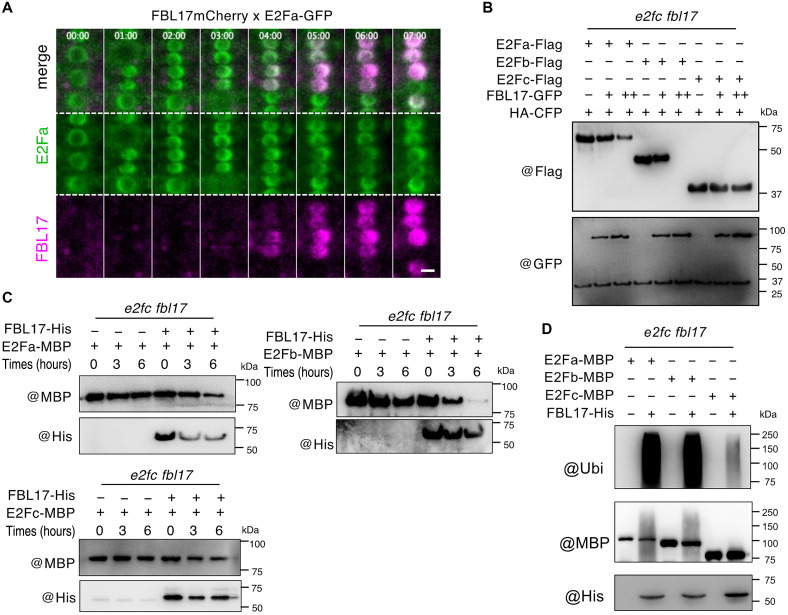
FBL17 promotes the polyubiquitylation and degradation of E2Fa/b, but not E2Fc. (**A**) Montage of a live imaging experiment of a root coexpressing FBL17-mCherry and E2Fa-GFP. Scale bar, 5 μm. (**B**) E2Fa-Flag, E2Fb-Flag, or E2Fc-Flag was expressed with (+) or without (−) FBL17-GFP in protoplasts of *e2fc fbl17*. The protein extracts were subjected to Western blotting. HA-CFP was used as the control of transfection efficiency. ++ indicates double amounts of plasmids. (**C**) In vitro degradation assays, E2Fa-MBP, E2Fb-MBP, or E2Fc-MBP was incubated with the total protein extracts from the callus of *e2fc fbl17* in the absence or presence of FBL17-His. (**D**) In semi–in vivo ubiquitylation assays, the E2Fa-MBP, E2Fb-MBP, or E2Fc-MBP proteins coupled with beads were incubated with the total protein extracts from callus of *e2fc fbl17* in a ubiquitylation buffer in the absence or presence of FBL17-His. MBP fusion proteins were pulled down and subsequently subjected to Western blotting.

With respect to the strong up-regulation of E2F target genes in *fbl17* [(*26*) and this work], we next investigated whether FBL17 interacts with one or more of these transcription factors. We first performed protein interaction assays in *Arabidopsis* protoplasts coexpressing FBL17-GFP with E2Fa-Flag, E2Fb-Flag, or E2Fc-Flag (Fig. 4E). In agreement with the Immunoprecipitation Mass Spectrometry (IP-MS) data, FBL17-GFP coimmunoprecipitated E2Fa and E2Fb but only poorly if any E2Fc. To test whether these interactions are direct, we performed pull-down assays in vitro. Recombinant E2F proteins fused to the maltose-binding protein (MBP) and His-tagged FBL17 (FBL17-His) were expressed in *Escherichia coli* and purified using affinity beads. FBL17-His could be pulled down by E2Fa-MBP and E2Fb-MBP but, to a lesser extent, with E2Fc-MBP (fig. S6A). To further test these protein interactions, we conducted yeast two-hybrid assays. We used *Arabidopsis* ASK1 as a positive control, since this protein is known to interact with numerous F-box proteins (*50*). These assays revealed that FBL17 interacts in yeast with both E2Fa and E2Fb, but not with E2Fc or with the E2F dimerization partner DPa (Fig. 4F). To confirm that these interactions also occur in plant cells, we performed split luciferase assays in *Nicotiana benthamiana* (Fig. 4G). The N-terminal luciferase (nLUC) domain was fused to FBL17, and the C-terminal luciferase (cLUC) domain was fused to all three E2Fs. A strong luminescence signal was detected by a highly sensitive cooled charge-coupled device (CCD) camera when nLUC-FBL17 was coexpressed with E2Fa-cLUC and E2Fb-cLUC, while the signal was considerably weaker with E2Fc-cLUC and absent with the cLUC domain alone. In addition, we performed bimolecular fluorescence complementation (BiFC) assays in *Arabidopsis* protoplasts (fig. S6B). Consistent with the results obtained by the split luciferase assays, a strong yellow fluorescent protein (YFP) fluorescence could be detected when nYFP-FBL17 was coexpressed with cYFP-E2Fa and cYFP-E2Fb, but not cYFP-E2Fc (fig. S6B). Furthermore, we performed live imaging experiments using roots of plants stably coexpressing FBL17mCherry and E2Fa-GFP and found that E2Fa is present in all nuclei except in those undergoing mitosis and that the cell cycle oscillations of FBL17 reveal an accumulation in the nuclei about 3 hours after cytokinesis, for a period of about 4 hours (Fig. 5A and movie S1). Also, FBL17 colocalizes with E2Fa, and the maximum intensity of FBL17 signal coincides with the minimum signal of E2Fa. Together, these data indicate that FBL17 specifically interacts with E2Fa and E2Fb and suggest that the F-box protein might be involved in their protein turnover.

To address this hypothesis, all three Flag-tagged E2Fs were coexpressed with FBL17-GFP in protoplasts derived from *e2fc fbl17* plants. We observed a strong reduction of E2Fa-Flag and E2Fb-Flag protein levels, only when they were coexpressed with FBL17-GFP in a dose-dependent manner (Fig. 5B). Conversely, the protein level of E2Fc-Flag was poorly affected by the expression of FBL17-GFP. Similar results were also obtained when we conducted in vitro degradation assays by incubating E2Fa-MBP, E2Fb-MBP, or E2Fc-MBP with protein extracts from the callus of *e2fc fbl17* in the absence or presence of FBL17-His (Fig. 5C). In the presence of FBL17, the degradation of E2Fa and E2Fb was more effective than that of E2Fc. Last, when incubated with total protein extracts from the callus of *e2fc fbl17*, E2Fa-MBP and E2Fb-MBP, but not E2Fc-MBP, were found ubiquitylated only in the presence of FBL17-His (Fig. 5D). From these results, we conclude that the overaccumulation of E2Fs in *fbl17* could be explained by an increase of their transcription (Fig. 3A) and a lack of degradation of some of them (Fig. 5) in this mutant. The fact that the severe cellular defects observed in *fbl17* are caused by E2Fc encourages us to further investigate its function.

### The capacity of E2Fc to bind chromatin is abrogated in the absence of FBL17

**Fig. 6.**
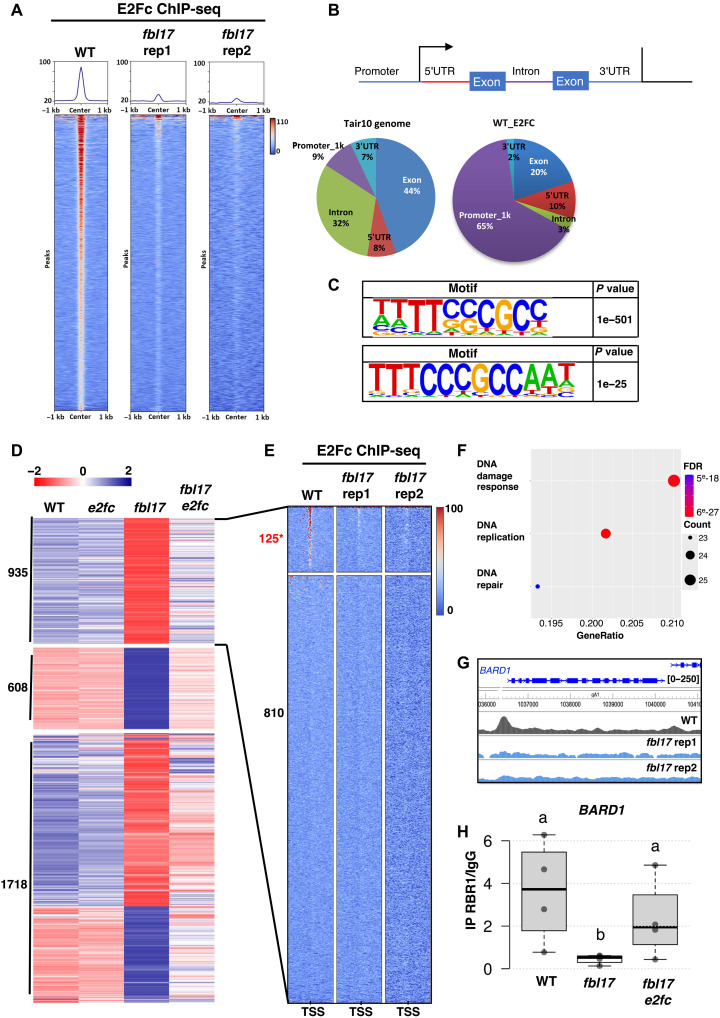
Chromatin binding of E2Fc is reduced in *fbl17*, leading to constitutive DDR genes activation. (**A**) Comparison of E2Fc enrichment in the WT and in the *fbl17* mutant in the ±1-kb region surrounding the E2Fc peaks. Two independent replicates are shown for *fbl17*. (**B**) Pie chart representation of the distribution of E2Fc peaks identified by ChIP-seq in different genomic regions in WT and *fbl17*. Transcription start site (arrow); 5′UTR, 5′ untranslated region; 3′UTR, 3′ untranslated region; transcription end site (vertical line). (**C**) HOMER motif search identified the canonical E2F-associated motif in WT and *fbl17*, with *P* values of 1 × 10^−501^ and 1 × 10^−25^, respectively. (**D**) Heatmaps displaying the expression profiles of three gene clusters in each genotype. The color scale corresponds to the *z*-score. (**E**) Comparison of E2Fc enrichment in WT and *fbl17* backgrounds for cluster 1 (*n* = 935). This cluster separates into two subclusters: one showing higher E2Fc binding in WT relative to *fbl17* replicates, representing direct targets of E2FC, and another with no detectable signal in any of the three samples, indicating that misregulation of the latter subcluster is not directly linked to E2FC activity. (**F**) Gene ontology enrichment analysis of the 125 genes found in the first subcluster. FDR, false discovery rate. (**G**) Screenshot of the *BARD1* locus from the Washu genome browser representing E2Fc ChIP-seq data in WT (black) and *fbl17* replicates (blue). The highlighted area corresponds to the region analyzed by ChIP-qPCR. (**H**) ChIP qPCR analysis of RBR1 binding to region highlighted on (G) in the WT, *fbl17*, and *fbl17 ef2c* lines. Data are relative enrichment compared to the IgY negative control. The vertical size of the boxes shows the interquartile range, and the whiskers correspond to 1.5× the interquartile range. The horizontal line corresponds to the median. Each individual replicate is represented by a dot. Different letters indicate statistically significant differences (Krukal-Wallis test, *P* > 0.05).

We next asked whether chromatin binding of E2Fc was affected by the *fbl17* mutation. To this end, we performed chromatin immunoprecipitation sequencing (ChIP-seq) assays with the pE2Fc:E2Fc-GFP construct expressed in *fbl17* or WT-like backgrounds. ChIP-seq analyses identified 1327 E2Fc targets in the WT (Fig. 6A). In agreement with previous findings (*33*), E2Fc was found to bind preferentially to the promoter region of genes, and the canonical E2F binding motif was significantly enriched in the E2Fc target sites (Fig. 6, B and C). Unexpectedly, we found that E2Fc binding to chromatin was markedly reduced in the *fbl17* background (Fig. 6A), although the E2F binding motif was still significantly enriched in the remaining target sites (Fig. 6B).

By performing RNA-seq analysis, we previously reported a large number of DE genes in *fbl17* with many cell cycle and DDR genes up-regulated, while a number of genes involved in primary metabolism are down-regulated (*26*). To have a more global view on the role played by E2Fc in the misexpression of those genes, we performed an RNA-seq analysis on 10-day-old *e2fc-1* and *fbl17-1 e2fc-1* seedlings in comparison with Col-0 WT seedlings as previously published (*26*). A total of 2013 up-regulated and 1250 down-regulated genes are found in *fbl17* compared to the WT (adj *P* < 0.05, |log_2_FC|>1) (*26*). Overall, genes up- or down-regulated in *fbl17* tended to return to WT or close to WT expression levels in the *fbl17 e2fc* double mutant (Fig. 6D). We next focused on genes that are respectively up-regulated in *fbl17* compared to Col0 and down-regulated in *fbl17 e2fc* compared to *fbl17* or down-regulated in *fbl17* compared to Col-0 and up-regulated in *fbl17 e2fc* compared to *fbl17* (Fig. 6D) and asked whether they could be direct E2Fc targets. The cluster of 608 genes that are down-regulated in *fbl17* and recover WT-like expression in *fbl17 e2fc* (Fig. 6D and fig. S7) shows enrichment in Gene Ontology terms related to metabolism and cell wall biogenesis. However, those genes are not E2FC direct targets.

E2Fc targets identified by ChIP-seq were significantly enriched among genes that were up-regulated in *fbl17* and recovered lower expression levels in *fbl17 e2fc* mutants (Fig. 6E). E2Fc binding to these genes was lost in the *fbl17* mutant (Fig. 6E and fig. S8). Gene ontology analysis of these 125 E2Fc targets revealed an enrichment in functions such as DDR, DNA replication, and DNA repair (Fig. 6F and also Fig. 3, A and B). Together, these results suggest that the constitutive activation of the DDR in the *fbl17* mutant is due to the absence of E2Fc binding to its targets. In the absence of E2Fc binding, the repression of these genes would be lost, leading to severe cell cycle progression defects and DDR. Because E2Fc functions as a repressor through its ability to recruit the RBR1 protein, we asked whether RBR1 binding could be lost on E2Fc targets in the *fbl17* mutant. Among E2Fc direct targets that are up-regulated in *fbl17* due to the absence of E2Fc binding, we found the *BARD1* gene (Fig. 6G). Note that E2FC binding is retained in the *fbl17* background for other genes (fig. S9). ChIP-qPCR analysis of RBR1 binding to the *BARD1* locus confirmed that RBR1 cannot bind to this target in the *fbl17* background but that RBR1 binding is restored in the *fbl17 e2fc* double mutant (Fig. 6H).

To explain the loss of E2Fc DNA binding in *fbl17*, we investigated whether its nuclear localization could be altered in this genetic background. Thus, we performed cellular fractionation experiments using the pE2Fc:E2Fc-GFP reporter lines. As cytoplasmic and nuclear markers, UGPase (UDP-glucose pyrophosphorylase) and histone H3 antibodies were used, respectively. The E2Fc-GFP signal was mainly detected in the nuclear fractions and even displayed an accumulation in the *fbl17-1* mutant background (fig. S10A). In addition, we imaged the subcellular localization of E2Fc-GFP in root tips of 10-day-old *fbl17-1* and WT-like backgrounds (fig. S10B). In the WT background, the E2Fc-GFP signal is localized in the nucleus, and also in the cytosol, in line with (*51*). In the *fbl17* mutant, we also observed the nuclear and cytoplasmic signals for E2Fc-GFP but with a notable reduced brightness for the nuclear signal. This suggests that the decreased DNA binding activity of E2Fc in *fbl17* is not the consequence of its impaired nuclear localization in this mutant background but rather linked to its altered activity.

## DISCUSSION

### Direct protein targets of FBL17 in plant cells

Ubiquitin-mediated proteolysis is a key mechanism underlying cell cycle control in all eukaryotes (*52*, *53*). In mammals, among several well-characterized E3 ubiquitin ligases, the F-box protein Skp2, as part of an SCF complex, plays a broad role in both cell cycle control and DNA damage by ubiquitylating a large number of regulatory proteins (*54*–*56*). Among these substrates, figure proteins involved in chromatin licensing and DNA replication, such as Cdt1 and Orc1p (*57*, *58*), cyclin E (*59*), the CKI protein p27 (*60*), transcriptional regulators including p130 (*61*), and proteins of the DDR (*62*), among many others. Despite such a large repertoire of substrates, mice that lack *Skp2* are viable, although with an overall reduced body size (*59*). This phenotype was attributed to the lack of degradation of CKI proteins as the size of the body and of different organs of the *Skp2 p27* double mutant mice were restored and even slightly larger than that of WT (*63*).

Among hundreds of F-box proteins identified in the *Arabidopsis* genome, *FBL17* appears to be the functional homolog of mammalian *Skp2* (*19*). For instance, both genes are regulated by E2F/DP transcription factors for periodic expression during the cell cycle (*20*, *43*, *64*), and both Skp2 and FBL17 proteins are phosphorylated by WEE1 (*65*) and are substrates of the APC/C for their degradation (*66*, *67*). However, contrary to *Skp2* null mice (*59*), the loss of function of *FBL17* in *Arabidopsis* is not viable, although few null mutant plants can be isolated, these exhibit major cell cycle defects and are fully sterile (*22*). It is generally assumed that KRP proteins are the main substrates of FBL17 [reviewed in (*19*)]. Hence, the steady-state level of KRP2 was found increased in *fbl17* mutants (*22*), and it has been shown that FBL17 is involved in the turnover of free, but not chromosomal bound, KRP4 to control cell size homeostasis in *Arabidopsis* (*25*). In addition, the overall phenotype of strong KRP2-overexpressing lines resembles *fbl17* mutant plants showing a drastic reduction in cell proliferation and suppression of endoreplication (*22*). However, strong biochemical evidence demonstrating that KRPs are direct substrates of FBL17 is still lacking, and at present, none of the *krp* mutation was reported to suppress the sporophytic phenotype of the *fbl17* null mutant. In addition, a ubiquitin-independent mechanism for *Arabidopsis* CKI INTERACTOR OF CDK 1 (ICK1)/KRP1 proteolysis has also been suggested (*68*). Notably, our IP-MS approach revealing the FBL17 interactome in young seedlings did not allow the detection of any KRP protein (Fig. 4A and table S2). Nevertheless, this might be explained by their low abundance and/or specific cell cycle expression patterns. Therefore, further studies are required to elucidate whether FBL17 really plays a direct role in the turnover of these cell cycle regulatory proteins.

Although the FBL17 interactome did not identify CKIs, it revealed several proteins of the RBR1-E2F/DP transcriptional module, suggesting that at least some of them might be targets of FBL17. At least in an *Arabidopsis* cell suspension, it was previously reported that RBR1 and E2F proteins are unstable being degraded by the proteasome during sucrose starvation (*69*). Moreover, under energy stress, E2Fa is phosphorylated by SnRK1.1 leading to its protein degradation (*70*), although the E3 ubiquitin ligase involved in this mechanism remains unknown. In our study, we found that RBR1, E2Fa, and E2Fc proteins all accumulated to higher levels in the *fbl17* null mutant (Fig. 4, B to D, and fig. S5). However, this effect could also largely depend on the transcriptional up-regulation of these genes in the *fbl17* mutant (Fig. 3A). Nevertheless, focusing on the E2Fs, we showed that E2Fa and E2Fb directly interact with FBL17 in both yeast and plant cells (Fig. 5, G and H, and fig. S6B). Moreover, both E2Fa/b proteins were ubiquitylated and degraded by FBL17 at least when transiently coexpressed in protoplasts and in in vitro degradation assays.

The protein accumulation level of E2Fa in *fbl17* remained nevertheless moderate, especially when compared to RBR1 (Fig. 4C and fig. S5). As the interaction of E2Fa with FBL17 is occurring at a specific cell cycle phase when the F-box gene is expressed (Fig. 5A), it is possible that the stabilization effects on the E2F protein is masked in protein extracts from entire seedlings. It is also conceivable that other E3 ubiquitin ligases are involved in the turnover of these proteins that would act redundantly to the SCF^FBL17^ ubiquitin ligase. In any case, even if the degradation of E2Fa and E2Fb by FBL17 may be relevant for cell cycle progression, this mechanism is unlikely to explain the severe phenotype of *fbl17* null mutants. If the accumulation of E2Fa and E2Fb in *fbl17* would be the direct cause of the strong up-regulation of cell cycle and DDR genes, one would expect that their mutations suppress the *fbl17* phenotype. However, this is not what was observed (Fig. 1). The phenotype of *fbl17 e2fa* mutant was even more affected than *fbl17* single mutant, and *fbl17 e2fb* could not be obtained as synthetically lethal, despite the fact that single *e2fa and e2fb* mutants are viable and exhibit a phenotype similar to WT plants.

### E2Fc a main repressor of cell cycle and DNA damage gene expression

In contrast to E2Fa and E2Fb, which are considered as transcriptional activators, it is assumed that E2Fc mainly functions as a transcriptional repressor (*71*–*74*). Hence, to repress cell cycle and other genes, E2Fc associates with MYB3Rs, RBR1, and other proteins that are part of a plant DREAM complex (*41*, *42*). E2Fc is known to be regulated at the posttranslational level (*73*) but is likely not a direct substrate of FBL17, as it does not clearly interact with and does not seem to be degraded by this F-box protein, at least in our experimental conditions (Figs. 3, E to H, and 4, B and C). However, a notable observation was that *e2fc* mutations largely abrogate the severe phenotype of the *fbl17* null mutant. To explain this phenotypic rescue, we initially thought that in the *fbl17* mutant background, E2Fc would lose its capacity as a repressor and would instead act as an activator to promote the strong transcriptional up-regulation of numerous genes and particularly those involved in cell cycle and DNA damage mechanisms. This hypothesis was also consistent with the fact that RBR1 was found phosphorylated in *fbl17* and thus unable to efficiently bind E2F proteins and repress transcription (*75*). However, while in a WT context, E2Fc binds a sequence similar to the consensus E2F site contributing to the repression of those genes [(*33*) and this work], and in the *fbl17* mutant background, this capacity was lost despite the strong accumulation of the E2Fc protein in this mutant. Thus, E2Fc is not directly responsible for the strong transcriptional up-regulation of these genes, but rather the reduced binding of E2Fc to chromatin and its inability to recruit RBR1 to its targets, as shown for *BARD1* (Fig. 6H), possibly explain the transcriptional misexpression of numerous E2F target genes (fig. S11). In addition, E2Fc not bound to chromatin in *fbl17* may titrate at least a fraction of RBR1, further alleviating the transcriptional repression of those genes. The mechanism by how E2Fc loses its capacity to bind chromatin in *fbl17* is still unclear. E2Fc is able to reach the nucleus (fig. S10), suggesting that other mechanisms at the posttranslational level, as for instance, the phosphorylation status of E2Fc, are at play, which will require further investigations.

Although it is tempting to speculate that the rescue of *fbl17* phenotypes by *e2fc* mutation is mainly due to the attenuation of the misexpression of cell cycle and DDR genes, other scenarios are also possible. For instance, it has been shown that E2Fc represses secondary cell wall biosynthesis genes (*76*). In line with this work, we found a number of metabolism and cell wall biogenesis genes down-regulated in *fbl17*, and their expression recovered to WT level in *fbl17 e2fc* (Fig. 6D and fig. S7). In any case, having viable *fbl17 e2fc* mutant plants now available opens the door to further investigate the function and the substrate repertoire of this essential F-box protein in cell cycle control, DNA damage, and beyond.

## MATERIALS AND METHODS

### Experimental models

*Arabidopsis thaliana* ecotype Columbia as well as *N. benthamiana* (for transient expression assays) and *Saccharomyces cerevisiae* (for yeast two-hybrid assays) were used in this study. The *Arabidopsis* mutants used in this study are as follows: *fbl17-1* (GABI_170E02), *e2fa-1* (MPIZ_244), *e2fa-2* (GABI_348E09), *e2fb-1* (SALK_103138), *e2fb-2* (SALK_120959), *e2fc-1* (GABI_718E1_025281), and *e2fc-2* (SAIL_1216_G10C1). The *fbl17-1 e2f* double mutants and reporter lines in the mutant background were generated by performing crosses and genotyping/sequencing of the resulting F2 and/or F3 progenies by PCR-based approaches. The list of primers used for cloning and genotyping is presented in table S3, as well as plasmids used for the different constructs. The cloning procedure of the constructs used is described below.

### Plasmid constructions

For yeast two-hybrid, the open reading frames of ASK1, E2Fa, E2Fb, E2Fc, DPa, and ASK1 were PCR amplified from first-strand cDNA and cloned in BamHI/XhoI digested pENTR3C-6HA except for ASK1, which was cloned in BamHI/NotI. The finished pENTRY constructs were recombined into pGAD424-GW (or pGBT9-GW in the case of FBL17). In all cases, splice variant 1 of The Arabidopsis Information Resource 10 (TAIR10) was used.

For coimmunoprecipitation assays, E2F coding sequences (CDSs) were cloned into pCambia2306 in Kpn I/Sal I sites to generate E2Fs-Flag constructs. For protein expression in *E. coli*, the E2F CDSs were cloned into pMALC2X at EcoR I/ Sal I sites to generate E2Fs-MBP constructs. For split luciferase assays, the CDS of E2Fs were cloned into pJW772 in Kpn I and Sal Ι sites. For BiFC assays, the E2F CDSs were cloned into pUC-SPYCE in Bgl II/BamH I sites. The constructs for FBL17-GFP, FBL17-His, nLuc-FBL17, FBL17-nYFP, and HA-CFP were cloned previously (*65*). For live microscopy colocalization, the genomic DNA of FBL17 was cloned into a pENTRY vector and then recombined into the pB7m34GW,0 by Gateway cloning.

### Plant growth conditions

For in vitro culture conditions, *Arabidopsis* seeds were surface-sterilized using ethanol and plated on MS agar [MES-buffered MS salt medium (Duchefa, Murashige and Skoog medium inc. vitamins/MES-MO255), 1% sucrose, and 0.8% agar (pH 5.7)]. Seeds were stratified for 2 days at 4°C in the dark and then transferred in 16-hour light/8-hour dark (20.5/17°C, 70% humidity) growth chamber, under fluorescent light (Osram Biolux 58 W/965). Plants grown on soil were under a 16-hour light/8-hour dark diurnal regime. For root cell microscopy and root elongation quantification, seeds were grown on MS agar plates (1% agar) positioned vertically in the growth chamber. For IP-MS and ChIP-seq experiments, seedlings were grown on MS medium with 1 ml of plant preservative mixture (PPM; Plant Cell Technology) per liter of medium to avoid the development of fungi from the seeds.

For IP-MS experiments, MLN-4924 treatment was performed on *Arabidopsis* seedlings grown for 9 days on MS agar plates and then transferred into liquid MS medium supplemented with either 25 μM MLN-4924 (an inhibitor of Nedd8 activating enzyme) or dimethyl sulfoxide (mock) overnight. Seedlings were snap-frozen in liquid nitrogen at day 10.

### Transient expression in protoplast

The isolation and transfection of *Arabidopsis* mesophyll protoplasts were performed as in (*77*).

### Leaf measurements and cellular analysis

For leaf area measurements, digital images were captured and processed using ImageJ 1.52p (http://imageJ.nih.gov/ij). Images of cell imprint were generated with a AxioImager Z1 inverted microscope (Zeiss) with Apotome. Cell size measurement was performed on the epidermal imprint images using ImageJ 1.52p (http://imageJ.nih.gov/ij) (*22*).

### Pollen viability assay

Pollen viability was assessed by mounting pollen grains in Alexander’s stain as described in (*20*) and observed by transmitted light microscopy using the AxioImager Z1 microscope (Zeiss).

### Root length analysis and confocal imaging

Root elongation analysis was performed as described in (*26*). Root tips observed under a confocal microscope (LEICA TCS SP8, Leica Microsystems) were prepared in propidium iodide (75 μg/ml). All microscopy images were treated and analyzed with the FIJI software (ImageJ 1.52p; http://imageJ.nih.gov/ij). Observations and/or dissection of siliques, anthers, and trichomes were done using a StereoMicroscope (Leica).

For live imaging experiments, 5-day-old seedlings were transferred to a glass-bottom plate (μslide, IBIDI), roots were covered with a block of MS 1% agar medium, and plants were acclimated overnight in the growth chamber. Confocal stacks covering half the root thickness (4-μm pinhole) were acquired every 15 min over an 8-hour period using a Nikon A1R+ inverted confocal microscope, equipped with a 40× oil-immersion objective. Laser lines used were 488 nm for GFP and 561 nm for mCherry. Hyperstacks were processed and analyzed using FIJI (*78*).

### Ploidy level measurement

Flow cytometry experiments were performed as reported in (*22*) and repeated at least three times using independent biological replicates. The Endoreduplication Index (EI) was calculated using the formula: EI = [0 · n2C + 1 · n4C + 2 · n8C + 3 · n16C + 4 · n32C + 5 · n64C]/[n2C + n4C + n8C + n16C+ n32C + n64C] (*79*).

### RNA extraction and RT-qPCR

Purification of total RNA from whole 10- and 20-day-old seedlings grown in vitro and qPCR conditions were performed as reported in (*22*). All primers used in RT-qPCR analyses are listed in table S3D.

### Analysis of RNA-seq data

Nucleic acid isolation, cDNA library preparation, and sequencing were performed as reported in (*26*). Single-end reads of RNA-seq samples were trimmed using Trimmomatic-0.38 with the parameters: minimum length of 30 bp; mean Phred quality score > 30; leading and trailing base removal with base quality < 5. The Hisat2.2.1 aligner was used to map the reads to the TAIR10 genome assembly. Raw read counts were then extracted using the featureCounts v2.0.0 utility from the Subread based on the TAIR10 genome assembly. Afterward, we used DESeq2 v1.34.0 to identify DE genes. R package pheatmap V1.0.12 and clusterProfiler V4.6.2 (*80*) were used to produce the heatmap for DE genes and gene ontology term analysis, respectively. The Venn diagram was generated using Evenn (*81*), and the odds ratio was calculated with the R package GeneOverlap.

### ChIP-seq and data analysis

ChIP-seq experiments were performed using 400 10-day-old plantlets. Plantlets were cross-linked in 1% (v/v) formaldehyde for 15 min, and cross-linking was quenched by adding glycine (125 mM final concentration) and incubating under vacuum for 5 min. Samples were ground in liquid nitrogen and resuspended in extraction buffer 1 [0.4 M sucrose, 10 mM tris-HCl (pH 8), 10 mM MgCl_2_, 5 mM β-mercaptoethanol, and 200 μl of a stock for 100-ml buffer (one tablet of cOmplete Mini, EDTA-free Protease inhibitor cocktail in 1 ml of sterile water)]. After filtration on 100-μm filters and centrifugation for 10 min at 1500*g*, pellets were resuspended and centrifuged two times for 5 min at 1000*g*, in extraction buffer 2 [0.25 M sucrose, 10 mM tris-HCl (pH 8), 10 mM MgCl_2_, 1% Triton X-100, 5 mM β-mercaptoethanol, and 200 μl of a stock (one tablet of cOmplete Mini, EDTA-free Protease inhibitor cocktail in 1 ml of sterile water) in 100 ml]. Last, nuclei were resuspended in nuclei lysis buffer [0.1% SDS, 50 mM tris-HCl (pH 8), and 10 mM EDTA (pH 8)]. Chromatin was sonicated for 10 min using Covaris S220 (peak power, 175; cycles/burst, 200; duty factor, 20). The sonicated chromatin was then immunoprecipitated (incubation at 4°C overnight on a rotating wheel) using 2 μl of anti-GFP antibodies (Abcam, ab290) (400 μl of sonicated chromatin + 600 μl of dilution buffer). Immunocomplexes were recovered with 40 μl of washed Dynabead protein A (Invitrogen, 10002D) for rabbit primary antibodies (incubation for 3 hours at 4°C with rotation). Beads were washed nine times with ChIP dilution buffer [1.1% Triton X-100, 1.2 mM EDTA, 16.7 mM tris-HCl (pH 8), 167 mM NaCl, and protease inhibitors] and incubated on a rotating wheel at 4°C for 5 min for each wash. ChIP DNA was eluted by two incubation steps for 15 min at 65°C with 200 ml of freshly prepared elution buffer (1% SDS and 0.1 M NaHCO_3_). Chromatin was reverse cross-linked by adding 16 μl of 5 M NaCl and incubated overnight at 65°C. The next day, chromatin was treated with 1 μl of ribonuclease: A + T1 (Ambion cocktail) for 20 min followed by incubation with 4 μl of Proteinase K (>600 U/ml), 8 μl of 0.5 M EDTA, and 16 μl of 1 M tris-HCl (pH 6.5) for 3 hours at 50°C. DNA was extracted with 450 μl of phenol:chloroform:indole-3-acetic acid (25:24:1) (pH 8). One milliliter of 100% ethanol was used to precipitate DNA after the addition of 1:10 NaOAc and 1 μl of GlycoBlue (15 mg/ml; centrifugation 10 min, 16,000*g* at 4°C), which was then resuspended in 10 μl of nuclease-free water. Libraries were then generated using a minimum of 2 ng of DNA with the NEBNext Ultra II DNA Library Prep Kit for Illumina (New England Biolabs). The quality of the libraries was assessed on a 2100 Bioanalyzer (Agilent), and the libraries were subjected to 75-bp high-throughput sequencing by NextSeq 500 (Illumina). Because of the limited amount of material (at most 1% of pE2Fc:E2Fc-GFP *fbl17^−/−^* seedlings can be isolated from the progeny of hemizygous plants), solely two biological ChIP-seq replicates were performed, but they were highly reproducible as evidenced by the scatterplots shown on fig. S12. For ChIP-seq data analysis, Trimmomatic-0.38 was used for trimming with the following parameters: minimum length of 30 bp; mean Phred quality score > 30; leading and trailing bases removal with base quality < 5. The reads were mapped onto the TAIR10 genome assembly (https://arabidopsis.org/) using Bowtie2.4.4 with mismatch permission of 1 bp. To identify significantly enriched regions, we used MACS version 2.2.7.1 with the default peak-calling parameters, *q* value threshold of 0.05 and extsize of 150, using inputs as negative controls. The heatmaps and peak profiles were generated by deepTools. Mapped BAM files were converted to bigWig format using deepTools and S3norm (*82*) normalized to configure the tracks in Integrated Genome Browser (IGB, version 10.0.1). Enriched motif analysis in E2Fc peaks was identified by HOMER (v4.11).

For ChIP-qPCR, the same procedure was followed, using 1.5 μl of anti-RBR antibodies (Agrisera, AS111627) or 1.5 μl of chicken immunoglobulin Y (IgY) as negative controls (Anticorps-en-ligne, ABIN376850) and 45 μl of agarose beads coupled with bovine anti-chicken IgY secondary antibody (Thermo Fisher Scientific, SA1-9588). Immunoprecipiated chromatin was diluted five times before qPCR that was performed using Roche LightCycler 480 SYBR Green I Master, according to the manufacturer’s instructions.

### Protein analysis and Western blotting

Total proteins were extracted from 10-day-old whole seedlings grown under in vitro conditions using denaturating buffer (*83*). Total protein extracts (5 to 15 μg) were separated on SDS–polyacrylamide gel electrophoresis gels and blotted onto Immobilon-P membrane (Millipore). Proteins were detected by using various antibodies, and Coomassie Brilliant Blue staining was used as a loading control.

### Cellular fractionation analysis

About 1 g of 10-day-old seedlings grown in vitro on MS-PPM was frozen and ground into a thin powder and resuspended into the extraction buffer [0.4 M sucrose, 10 mM tris-HCl (pH 7.5), 10 mM MgCl_2_, 2.5 mM dithiothreitol (DTT), and 1× Protease inhibitor cocktail]. The liquid suspension was filtered twice through a Miracloth filter, and a fraction of the suspension was collected as the total protein extract. After centrifugation (20 min at 3200*g*), a fraction of the supernatant was collected as the cytosolic fraction. The pellet was resuspended in washing buffer [0.25 M sucrose, 1% Triton X-100, 10 mM tris-HCl (pH 7.5), 10 mM MgCl_2_, 2.5 mM DTT, and 1× Protease inhibitor cocktail] and centrifuged (10 min at 16,000*g*), and the supernatant containing the burst organelles was discarded. The nuclei pellet was washed until white appearance. The clean pellet was resuspended into washing buffer and loaded onto a coat of a high-sucrose buffer [2.5 M sucrose, 0.15% Triton, 10 mM tris-HCl (pH 7.5), 2.5 mM MgCl_2_, 2.5 mM DTT, and 1× Protease inhibitor cocktail]. After 1-hour centrifugation (16,000*g*), nuclei have pelleted through the sucrose gradient, and they were resuspended in the nuclei storage buffer [0.4 M sucrose, 6.25% glycerol, 20 mM tris-HCl (pH 7.5), 2.5 mM MgCl_2_, and 1× Protease inhibitor cocktail] and stored at −80°C as the nuclear fraction. Last, the different fractions were extracted in 1× Laemmli buffer to quantify total proteins.

### IP and MS analyses

For IP of FBL17-GFP, 0.33 g of frozen plant material (10-day-old seedlings previously treated in 25 μM MLN-4924 for 16 to 24 hours) was ground to a fine powder with mortar and pestle, resuspended in 3 volumes of IP extraction buffer [50 mM tris base, 150 mM NaCl, 5 mM MgCl_2_, 50 μM MG132, 10% glycerol, 3 mM DTT, 60 mM p-glycerophosphate, 0.1% NP-40, 0.5 mM NaF, 1 mM AESBF, and 1 cOmplete Protease Inhibitor Cocktail (Roche)].

For the formaldehyde IPs, 50 mM HCL-free tris was used in the buffer with a final formaldehyde concentration of 0.375%. An additional step of glycine 200 mM final was added for the quenching step of the bridging agent. Insoluble material was removed by centrifugation (twice, 15 min, 12,000*g*, 4°C). Identical amounts of crude extracts were incubated with 50-μl anti-GFP magnetic beads (Anti-GFP Microbeads from μMACS GFP Isolation kit, Miltenyi) for 30 min at 8 rpm in the cold room. Immune complexes were washed four times in the crude extract buffer, and purified protein complexes were eluted from the beads in the elution buffer following the manufacturer’s instructions.

Eluted proteins were reduced (5 mM DTT), alkylated (10 mM iodoacetamide), and digested with 300 ng of tryspin. Generated peptides were analyzed by nano-scale liquid chromatography–tandem mass spectrometry (nanoLC-MS/MS) on a reversed phase nanoElute 2 coupled to a TIMS-TOF Pro 2 mass spectrometer (Bruker Daltonik GmbH) using a data-independent acquisition and parallel accumulation-serial fragmentation. Peptides were separated on an IonOpticks Aurora Elite column (25 cm by 75 μM, 1.7-μm particle size, and 120-Å pore size; AUR3-15075C18-CSI) with 45-min gradients. Data were searched against the TAIR database using DIANN 1.9 software with a library-free approach. Prostar software (1.34.5) was used for the statistical analyses of the intensities. Imputation of missing values was based on the 0.9% det quantile. A LIMMA statistical test and a Benjamini-Hochberg correction were used to generate log_2_(FC) and adjusted *P* values.

### Coimmunoprecipitation assays

E2Fa-Flag, E2Fb-Flag, or E2Fc-Flag was coexpressed with FBL17-GFP in *Arabidopsis* protoplasts. Total proteins were extracted using IP buffer containing 100 mM tris-HCl (pH 7.0), 150 mM NaCl, 0.1% NP-40, 1 mM phenylmethylsulfonyl fluoride (PMSF), 100 μM MG132 (MedChemExpress), and protease inhibitor cocktail (Sigma-Aldrich). The supernatant was precipitated by GFP-Trap (Chromotek) at 4°C for 3 hours. The beads were washed three times with washing buffer containing 100 mM tris-HCl (pH 7.0), 150 mM NaCl, 0.1% NP-40, and 2 mM PMSF and then boiled in 5× SDS loading buffer at 100°C for 5 min. The proteins bound to beads were subjected to Western blotting using anti-GFP (1:4000; Thermo Fisher Scientific) or anti-FLAG (1:4000; Sigma-Aldrich) antibodies.

### Pull-down assays

The E2Fa-MBP, E2Fb-MBP, or E2Fc-MBP proteins coupled to Dextrin beads were incubated with FBL17-His proteins in a binding buffer [1% Triton X-100, 150 mM NaCl, 2 mM EDTA, and 50 mM tris-Cl (pH 8.0)] at 4°C for 2 hours. The beads were then washed five times with phosphate-buffered saline buffer [140 mM NaCl, 2.7 mM KCl, 10 mM Na_2_HPO_4_, and 1.8 mM KH_2_PO_4_ (pH 7.4)] containing 1% Triton X-100. The proteins were eluted by incubating beads with 5× SDS loading buffer at 100°C for 5 min. Both the input and pull-down samples were subjected to Western blotting using anti-His (1:3000; Biodragon) or anti-MBP (1:3000; Biodragon) antibodies.

### Yeast two-hybrid

The yeast two-hybrid analysis was performed following the Clontech Matchmaker Two-hybrid manual (Takara Bio). The Gateway-converted binding-domain vectors (pGBT9) and the activation-domain vector (pGAD424) were used in (*84*). Yeast strain PJ69-4A. The transformation protocol followed is reported in (*85*).

### Split luciferase assay

The corresponding plasmids were introduced into *Agrobacterium tumefaciens* strain GV3101 and then infiltrated into *N. benthamiana* leaves. Two days after infiltration, 1 mM luciferin (GOLDBIO) was administered on the leaves, and images were captured using the Lumazone imaging system equipped with a 2048B CCD camera (Roper).

### BiFC assays

The corresponding plasmids were transfected into *Arabidopsis* protoplasts as indicated in (*86*). YFP fluorescence was detected using a confocal laser scanning microscope (TCS SP8, Leica) with excitation at 488 nm, and emission was captured between 500 and 535 nm.

### Semi–in vivo ubiquitination assays

The E2Fa-MBP, E2Fb-MBP, or E2Fc-MBP proteins coupled with Dextrin beads were incubated with the total protein extracts from the callus of e2fc fbl17 in a ubiquitination buffer [25 mM tris-HCl (pH 7.5), 10 mM MgCl_2_, 10 mM NaCl, 10 mM adenosine triphosphate (ATP), 5 mM DTT, and 100 μM MG132] in the absence or presence of FBL17-His for 3 hours. Then, the beads were washed three times with radioimmunoprecipitation assay buffer. Last, the beads were subjected to Western blotting using anti-ubiquitin, anti-His, or anti-MBP antibodies.

### Coexpression assays

The plasmids expressing E2Fa-Flag, E2Fb-Flag, E2Fc-Flag, HA-CFP, and/or FBL17-GFP were cotransfected into the protoplasts of e2fc fbl17. After 10-hour incubation, the total proteins were extracted using extraction buffer [100 mM tris-HCl (pH 7.0), 150 mM NaCl, 0.1% NP-40, 1 mM PMSF, and 1× protease inhibitor cocktail] and were subjected to Western blotting using anti-GFP or anti-flag antibodies.

### In vitro degradation assays

The E2Fa-MBP, E2Fb-MBP, or E2Fc-MBP proteins were incubated with the total protein extracts from the callus of e2fc fbl17 in a proteolysis buffer [25 mM tris-HCl (pH 7.5), 10 mM MgCl_2_, 10 mM NaCl, 10 mM ATP, and 5 mM DTT] in the absence or presence of FBL17-His at room temperature for different times. The reactions were stopped by adding the 5× SDS loading buffer and boiled at 100°C for 5 min. The samples were subjected to Western blotting using anti-MBP or anti-His antibodies.

### Quantifications and statistical analysis

All statistical analyses were performed in RStudio (Mann-Whitney tests), and results are listed in table S1.
