# Supplementary material for: Tuning of the RBR1-E2F/DP transcriptional module by the F-box protein FBL17
Source: Sci Adv. 2026 Feb 18;12(8):eadz2439. doi: 10.1126/sciadv.adz2439 (PMC12915602; doi:10.1126/sciadv.adz2439)
Supplement: Supplementary file 1 — Figs. S1 to S12 [file sciadv.adz2439_sm.pdf]

Science Advances
AAAS

Supplementary Materials for

Tuning of the RBR1-E2F/DP transcriptional module by the F-box protein FBL17

Juliette Espanet *et al.*

Corresponding author: Sandra Noir, sandra.noir@ibmp-cnrs.unistra.fr;
Pascal Genschik, pascal.genschik@ibmp-cnrs.unistra.fr

Sci. Adv. **12**, eadz2439 (2026)
DOI: 10.1126/sciadv.adz2439

The PDF file includes:

Figs. S1 to S12

Other Supplementary Material for this manuscript includes the following:

Tables S1 to S3
Movie S1

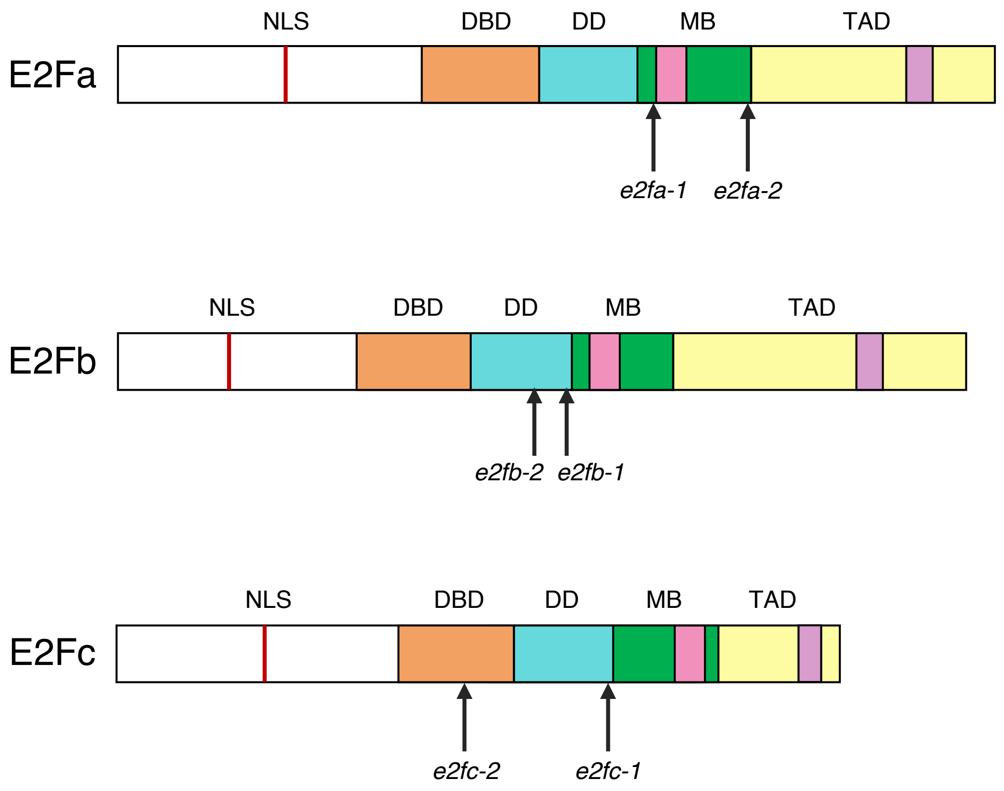

Fig. S1. T-DNA insertion lines of the three canonical Arabidopsis *e2f* mutants
Representation of the three canonical E2F protein sequences with their different protein domains indicated in colour; DNA-binding domain (DBD; orange), a heterodimerization domain composed of the dimerization domain (DD; blue) and the Marked box domain (MB; green), and finally a trans-activation domain (TAD; yellow). Rb-binding domain (purple) in the TAD and additional putative Rb binding domains (pink) in the Marked box are also indicated. N-terminal nuclear localization signals (NLS) are indicated by a red line. Locations of the respective T-DNA insertions are indicated by arrows.

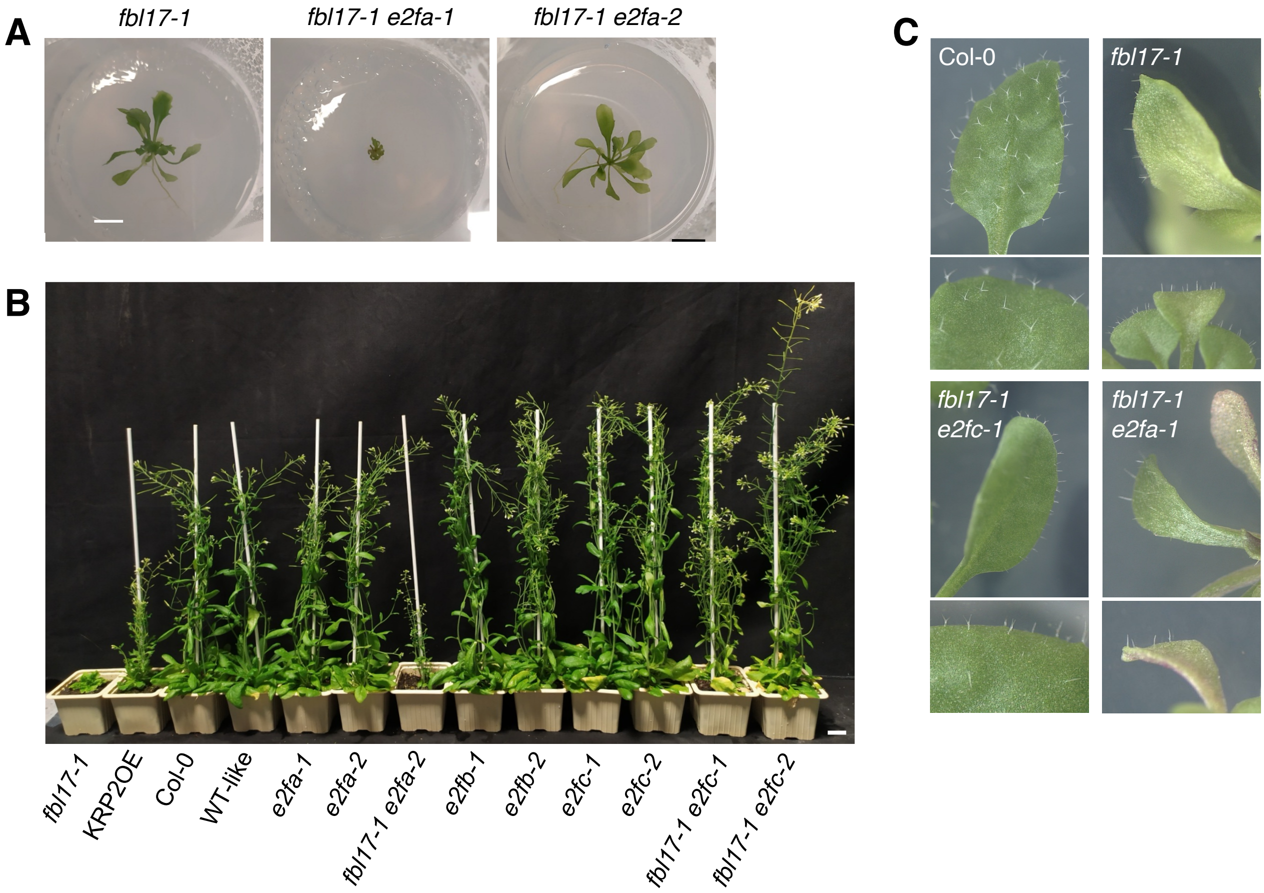

Fig. S2. Phenotypes of mutants grown *in vitro and* on soil

(A) Representative pictures of *fbl17-1* and the double mutants, *fbl17-1 e2fa-1* and *fbl17-1 e2fa-2* rosettes *in vitro* grown on MS medium for 30 days. Scale bar = 1 cm.

(B) Representative pictures of 8-week-old flowering plants of the different genotypes; WT-like plants from *fbl17+/-* progeny. Scale bar = 2 cm.

(C) Trichomes observed on the first pair of 20-day-old plants of the indicated genotypes.

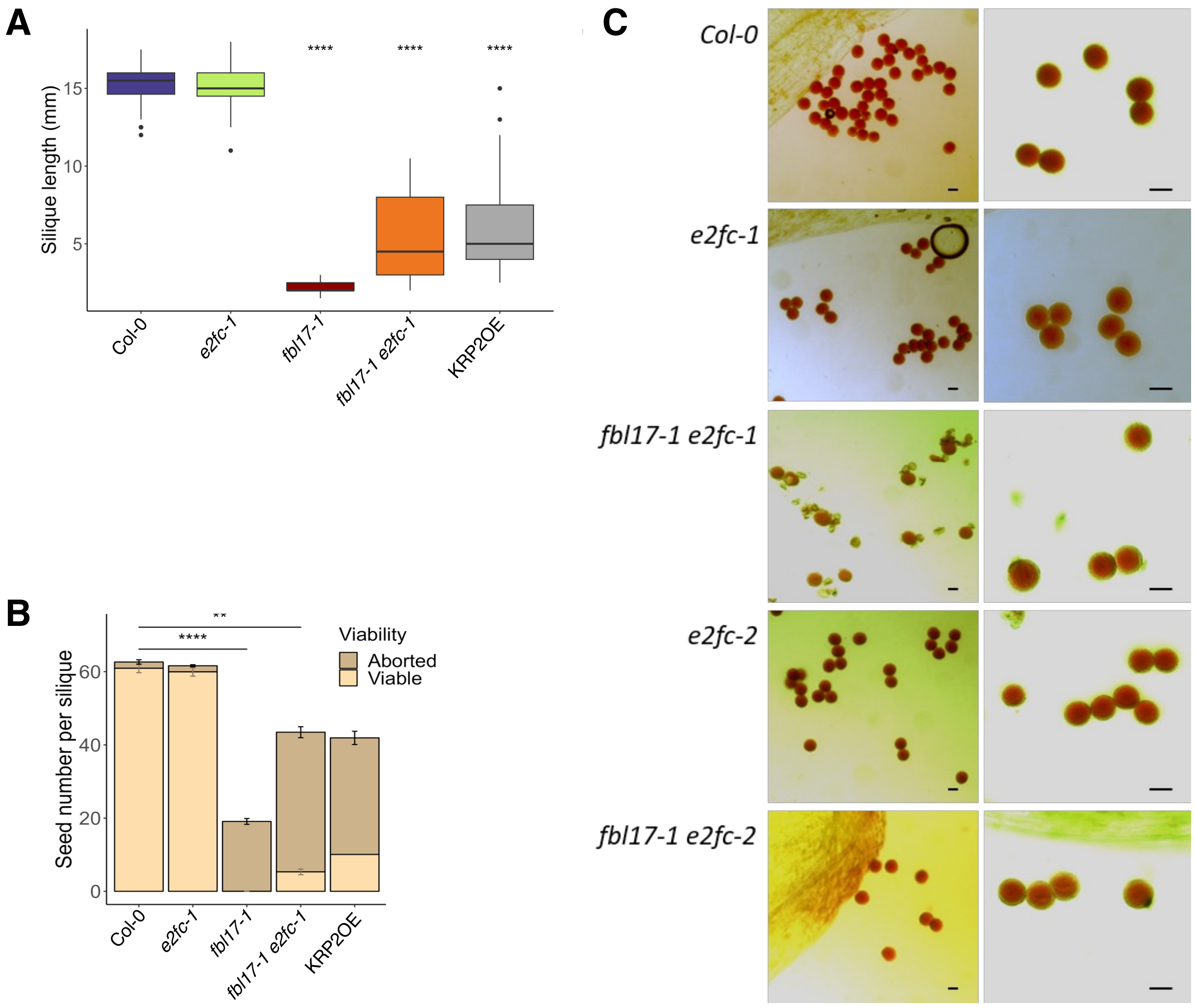

Fig. S3. Characterization of *fbl17 e2fc* mutant fertility

(A) Graphs showing the silique length of the indicated genotypes.

(B) Number of seeds per silique, including aborted seeds indicated in brown. Error bars indicate standard error variation from 3 biological replicates. Asterisks indicate significant difference between the different genotypes and Col-0 plants between three biological replicates : **P<0,01, ****P<0,001 by Wilcoxon-Mann-Whitney test. Complete statistical analyses are given in Supplemental Tables 1L-M.

(C) Pollen viability was assessed by using Alexander staining. Pollen grains from 5 distinct flowering plants per genotype were collected for analysis. The pictures illustrate the pollen viability observed for each genotype. Scale bar = 10 µm.

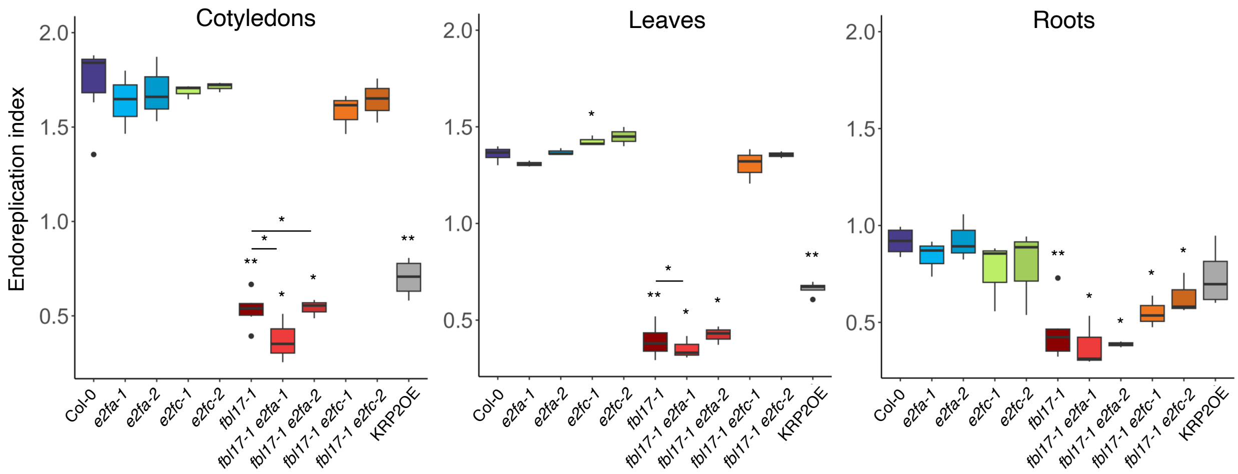

Fig. S4. Endoreplication rate of *fbl17 e2fc* mutants

Endoreplication index (EI) calculated with the DNA ploidy results presented in the figure 2F. The EI value represents the mean number of endoreplication cycles per nucleus. Asterisks indicate significant difference between the corresponding genotypes and Col-0 plants from each biological replicates ($3 \le n \le 6$) : *<P 0.05 and **P<0.01 by Wilcoxon-Mann-Whitney test. Complete statistical analyses are given in Supplemental Table 1N.

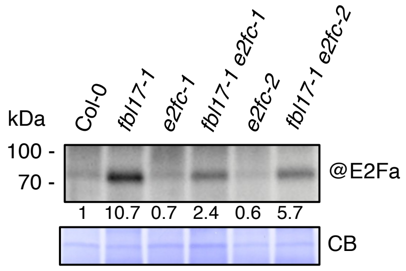

Fig. S5. Biological replicate for the accumulation of E2Fa in *fbl17*, *e2fc* and *fbl17 e2fc* mutants

Immunoblot analysis of protein extracts from 10-day-old seedlings of the indicated genotypes to monitor E2Fa protein level. Coomassie blue (CB) staining was used as loading control.

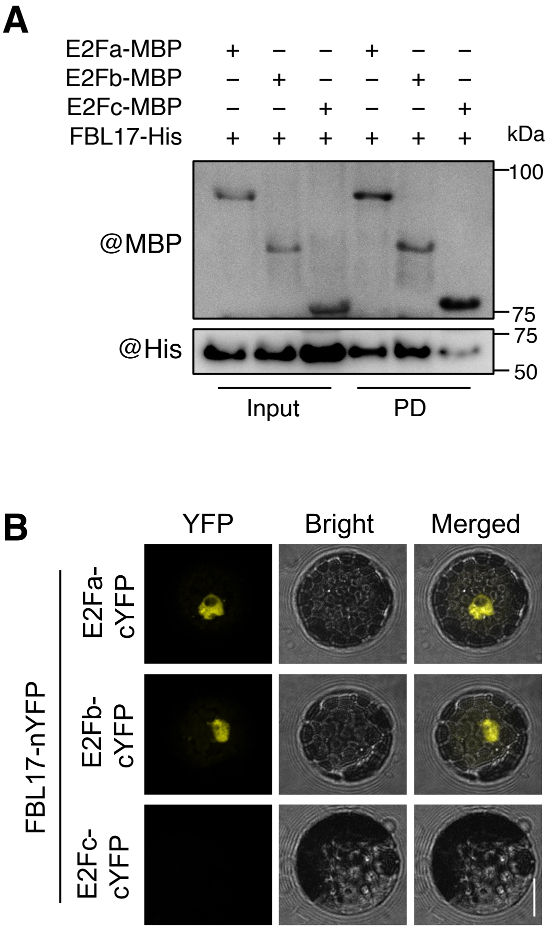

Fig. S6. Pull-down and BiFC interaction assays

(A) *In vitro* pull-down assays, the E2Fa-MBP, E2Fb-MBP or E2Fc-MBP coupled with beads were used to pull down FBL17-His. Both the input and pull-down samples were subjected to western blotting.

(B) The N-terminal YFP (nYFP) was fused to FBL17, while the C-terminal YFP (cYFP) was fused to E2Fs. In BiFC assay, FBL17-nYFP was co-expressed with E2Fa-cYFP, E2Fb-cYFP, or E2Fc-cYFP in Arabidopsis protoplasts. The YFP fluorescence detected by a confocal microscopy indicates interaction. Scale bar, 100 μm.

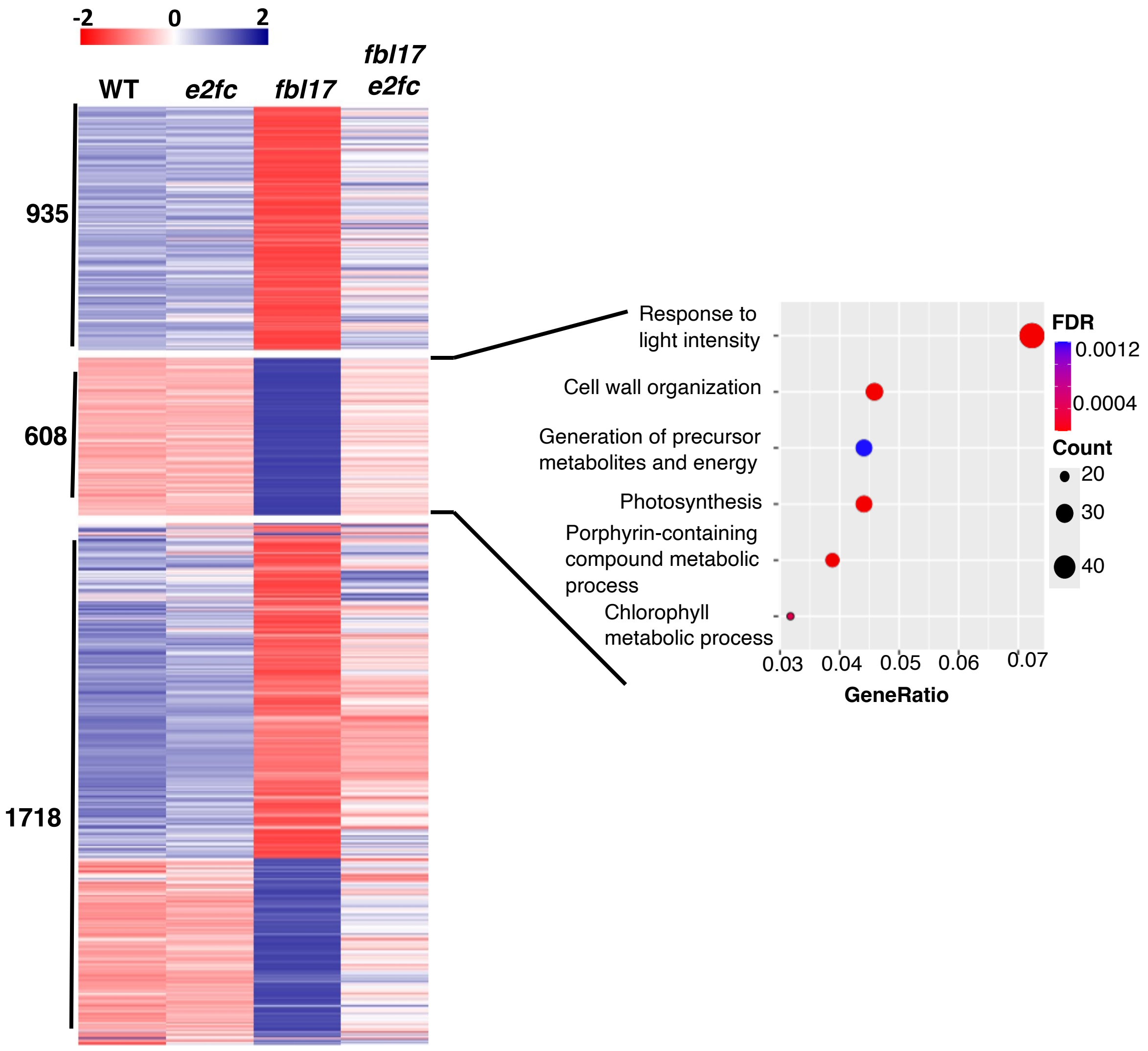

Fig. S7. Heatmaps showing the expression of three gene clusters in WT, *e2fc, fbl17 and* the *e2fc fbl17* double mutant

The colour scale corresponds to the Z-score. The second cluster, comprising genes that are down-regulated in *fbl17* and recover WT-like expression in *fbl17 e2fc* does not contain E2FC direct targets. Gene ontology enrichment analysis of these 608 genes revealed enrichment in GO terms related to metabolism and cell wall biogenesis.

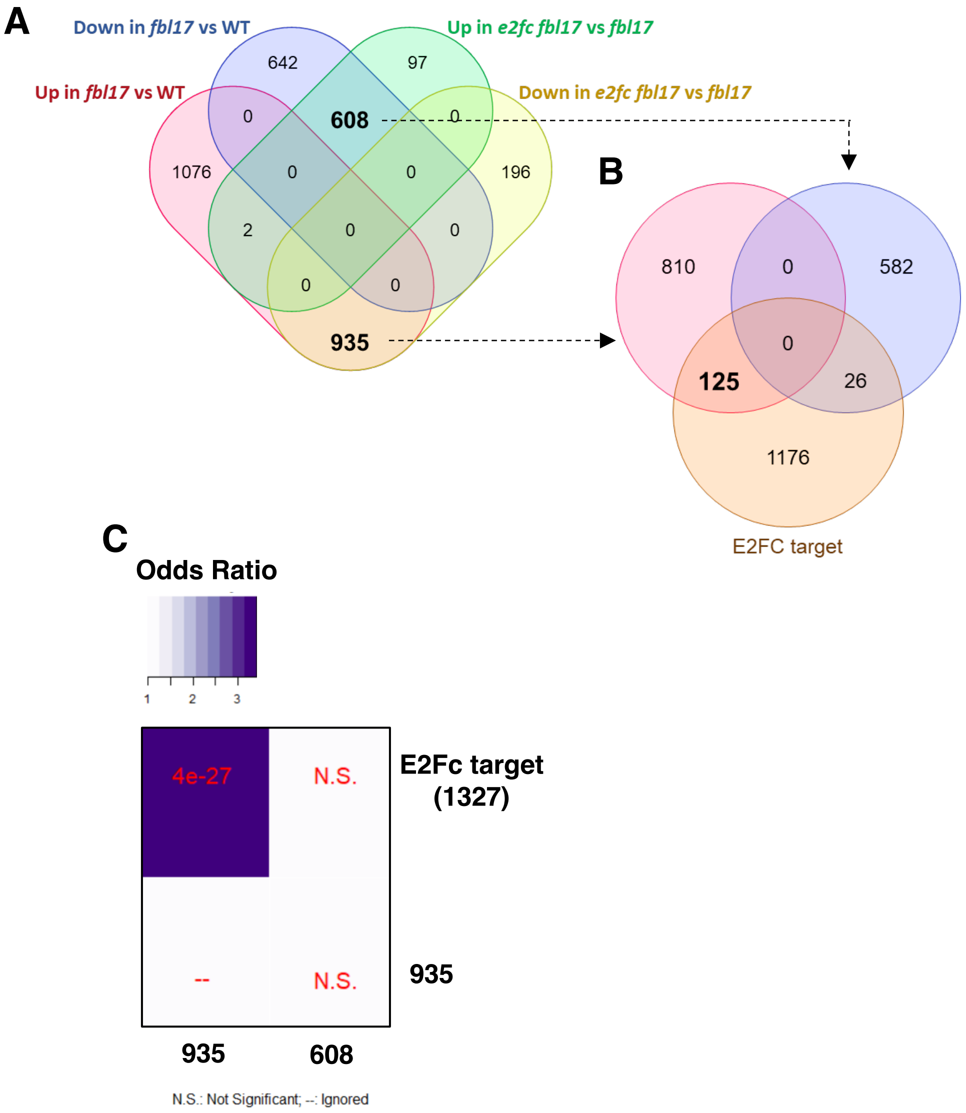

Fig. S8. E2Fc targets are upregulated in the *fbl17* mutant

(A) Venn diagram showing the overlap between DEG in *fbl17* compared to the WT, and DEG in *e2fc fbl17* compared to *fbl17*. Approximatively half of upregulated genes in *fbl17* are down-regulated in *e2fc fbl17* and reciprocally, half of genes down-regulated in *fbl17* are upregulated in *e2fc fbl17*.

(B) Venn diagram comparing the list of genes whose expression reverts towards their expression in the WT with the list of E2Fc direct targets. Most DEGs that are direct targets of E2Fc are upregulated in *fbl17* and return to lower expression levels in *e2fc fbl17*.

(C) Odds ratio (Fisher's exact test) showing that E2F targets are significantly enriched only in the category of genes that are up-regulated in *fbl17* and return to lower expression levels in *e2fc fbl17*.

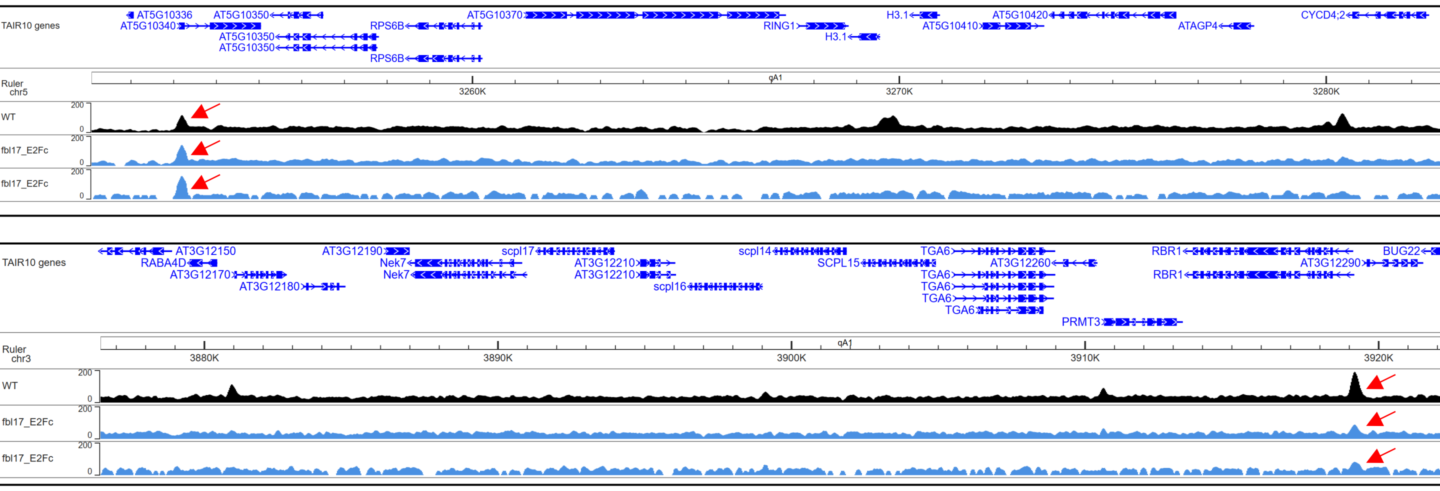

Fig. S9. Genomic loci in which E2Fc binding is retained in the *fbl17* background Screenshots showing E2Fc binding in the WT and in the *flb17* mutant. Most E2Fc binding is lost in the *fbl17* mutant, but some peaks remain detectable: red arrows indicate peaks that are retained in *fbl17*.

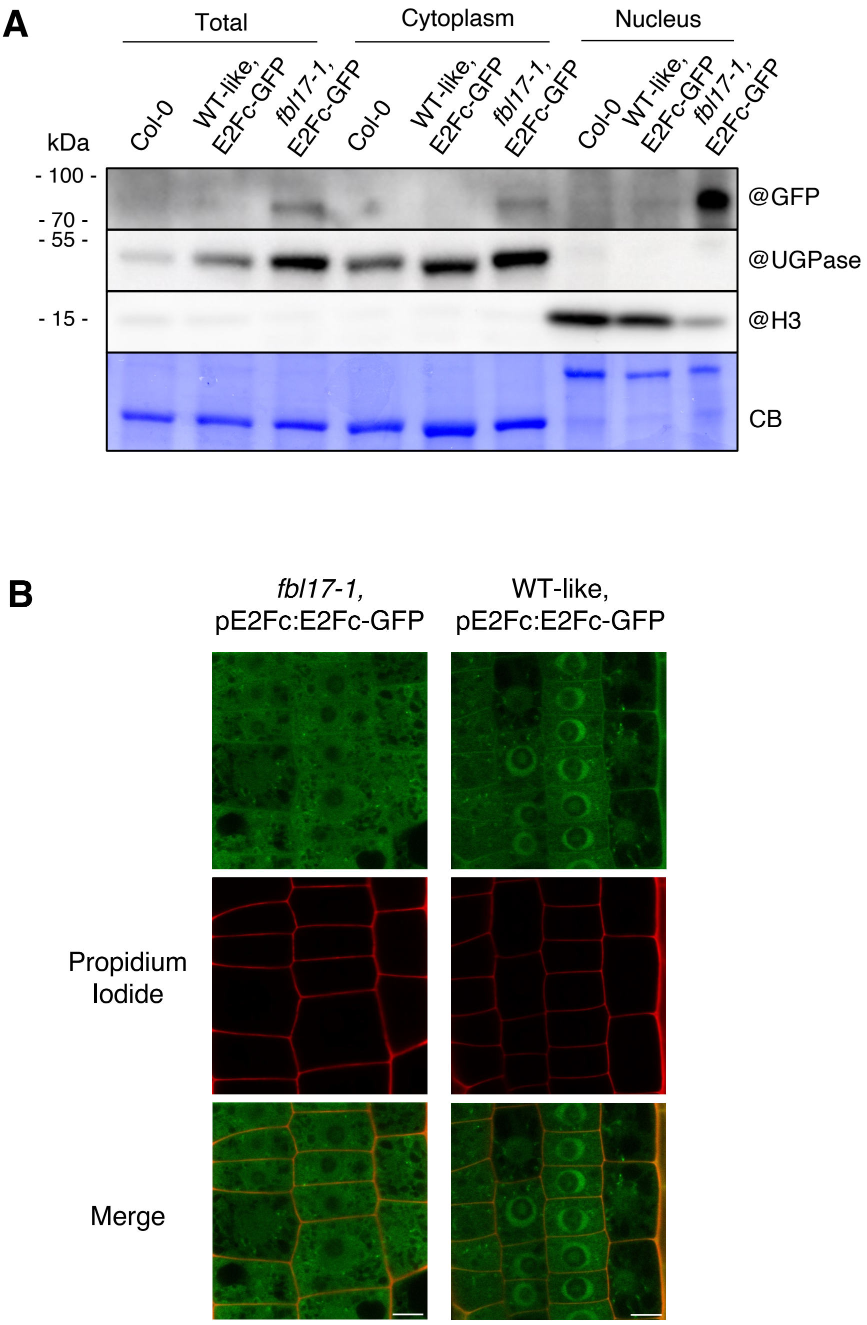

Fig. S10. E2Fc sub-cellular distribution in *fbl17* mutant line

(A) Western-blot analysis of E2Fc-GFP protein from total, cytosolic and nuclear fractions isolated from 10-day-old *fbl17-1*, pE2Fc:E2Fc-GFP and WT-like, pE2Fc:E2Fc-GFP seedlings. The H3 and UGPase antibodies are used for nuclear and cytoplasm controls, respectively. Two biological replicates gave the same tendency. Col-0 is used as a negative control and Coomassie blue (CB) staining is used as a loading control.

(B) Representative images showing E2Fc subcellular localization in roots of 10-day-old plants of the indicated genotypes. Scale bar = 10 μm.

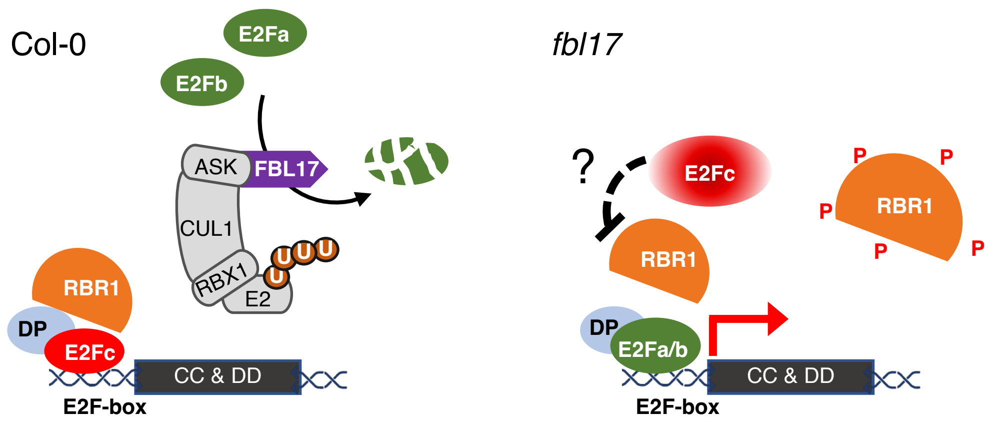

Fig. S11. Speculative model for the role of FBL17 in regulating the RBR1/E2F transcriptional module

In Col-0, FBL17 interacts with E2Fa/b and triggers their degradation, most likely in a cell cycle dependent manner. E2Fc in association with RBR1 and other factors represses cell cycle (CC) and genes involved in DNA damage (DD). In the *fbl17* mutant, the protein level of all three E2Fs accumulate to a higher level. In this genetic background, E2Fc lost its capacity to bind chromatin and to repress the transcription of E2F target genes. In addition, DNA-unbound E2Fc may also titrate the unphosphorylated and active pool of RBR1. In the absence of repression, E2Fa and/or E2Fb or other transcription regulators would activate CC and DD genes leading to constitutive DDR and cell death.

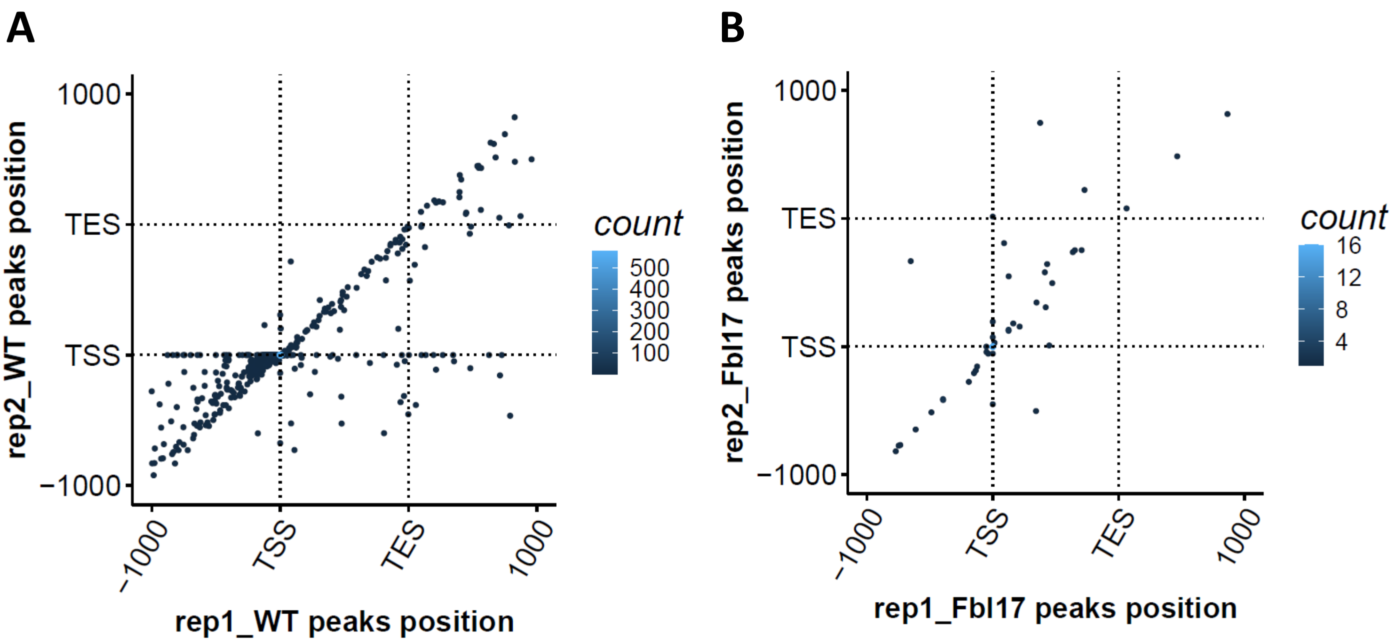

Fig. S12. Graphs showing the position of peaks identified in the two ChIP-seq replicates performed in WT and *fbl17* lines. Each dot represents one peak.
